# A Comprehensive Review of Nanoparticles: From Classification to Application and Toxicity

**DOI:** 10.3390/molecules29153482

**Published:** 2024-07-25

**Authors:** Furkan Eker, Hatice Duman, Emir Akdaşçi, Ecem Bolat, Sümeyye Sarıtaş, Sercan Karav, Anna Maria Witkowska

**Affiliations:** 1 Department of Molecular Biology and Genetics, Çanakkale Onsekiz Mart University, Çanakkale 17000, Türkiye; furkan.eker@stu.comu.edu.tr (F.E.); hatice.duman@comu.edu.tr (H.D.); emirakdasci@gmail.com (E.A.); ecemmbolatt@gmail.com (E.B.); sumeyyesaritas@stu.comu.edu.tr (S.S.); 2 Department of Food Biotechnology, Medical University of Bialystok, 15-089 Bialystok, Poland

**Keywords:** nanoparticles, nanotechnology, industrial applications, toxicity, organic nanoparticles, carbon-based nanoparticles, inorganic nanoparticles

## Abstract

Nanoparticles are structures that possess unique properties with high surface area-to-volume ratio. Their small size, up to 100 nm, and potential for surface modifications have enabled their use in a wide range of applications. Various factors influence the properties and applications of NPs, including the synthesis method and physical attributes such as size and shape. Additionally, the materials used in the synthesis of NPs are primary determinants of their application. Based on the chosen material, NPs are generally classified into three categories: organic, inorganic, and carbon-based. These categories include a variety of materials, such as proteins, polymers, metal ions, lipids and derivatives, magnetic minerals, and so on. Each material possesses unique attributes that influence the activity and application of the NPs. Consequently, certain NPs are typically used in particular areas because they possess higher efficiency along with tenable toxicity. Therefore, the classification and the base material in the NP synthesis hold significant importance in both NP research and application. In this paper, we discuss these classifications, exemplify most of the major materials, and categorize them according to their preferred area of application. This review provides an overall review of the materials, including their application, and toxicity.

## 1. Introduction

Nanoparticles (NPs) are nanostructures that have a size of up to 100 nanometers applied to all directions (x, y, and z). These particles are unique due to their small size and configurable functions with surface molecules. Owing to these, their small particle sizes and large surface areas alter their molecular interactions, creating new areas of application [1]. The characteristics of the particles and their structures are the key parameters that determine the functionality, activity, and utility of the NPs. For instance, the size and the shape of the NP can determine the modification possibilities, optical properties, and its entry to cells, which is important for applications like cancer treatment and imaging studies [2]. Additionally, the surface charge of the NP is a major factor that influences the interaction in the environment, affecting not only cellular interaction but also the toxicity potential along with the characteristics of the material [3].

**Figure 1:**
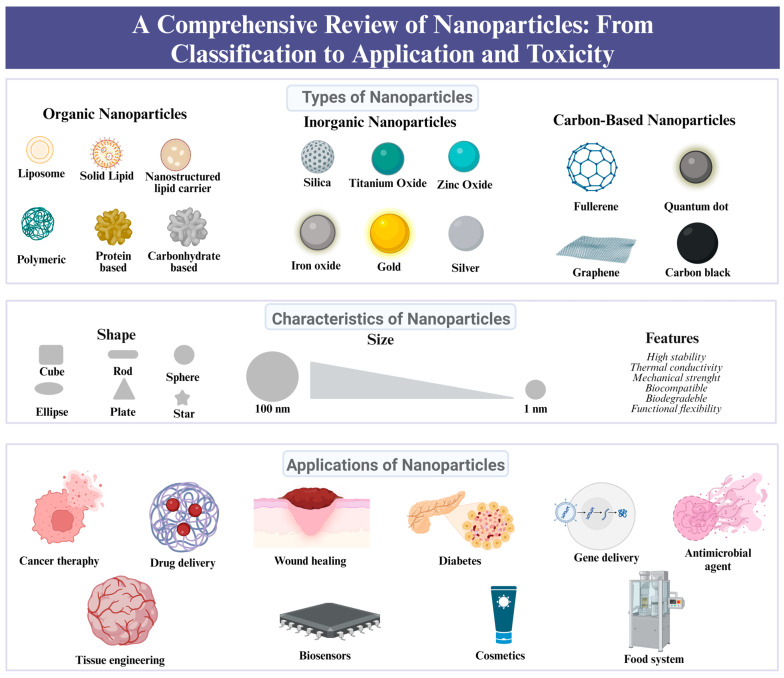
Representative scheme of NPs.

All of these factors impact the application of the NP and lead to distinctiveness in their use. In the current literature, many reviews evaluate the detailed application of NPs in specific areas, such as industry [4], food products [5], drug delivery [6], antimicrobial activity [7], imagining [8], therapeutics [9], and so on. When these applications are inspected, different types of NPs are predominant in certain areas. In other words, many attributes of the NPs affect their activity and shift their preference to certain specialties. The synthesis methods, shape, and size of the NPs can significantly change the efficiency and activity of the particle. Considering all these factors, the chosen material is also a primary variable that determines the role, and especially the type, of NP. Based on the material, NPs are classified into three main types—inorganic, carbon-based, and organic—and subdivided into different kinds such as metallic, ceramic, polymeric, and lipid-based due to their distinct characteristics, size, and shape [10] (Figure 1).

The primary goal of this article is to analyze nanoparticle categories based on their main structures, examine their features and applications, investigate their use with other NPs and nanocompounds, and cover the toxicity of commonly used production materials. Thus, we assessed the classification of NPs into three bases that characterize the material’s main structure. We covered these three groups and explained the properties and applications of the majority of the top sub-materials. Finally, we briefly examined the toxicity of various elements commonly used in NP synthesis.

## 2. Classification of NPs

**Figure 2:**
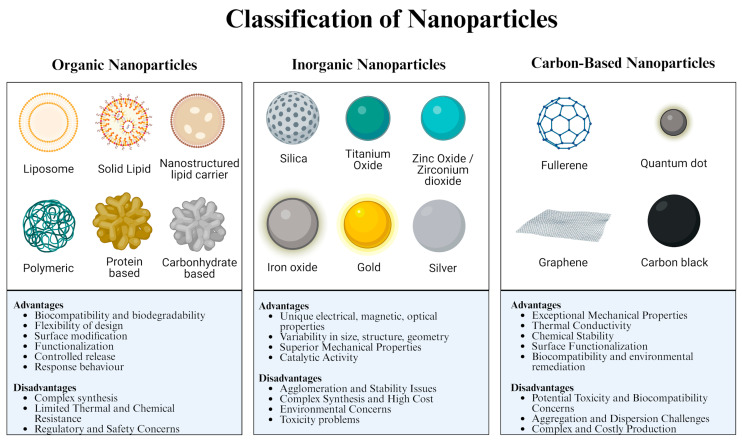
Classification of nanoparticles [11,12].

As addressed, NPs are classified into three categories, organic, inorganic, and carbon-based, with each of these having their advantages and disadvantages (Figure 2).

### 2.1. Inorganic NPs

**Figure 3:**
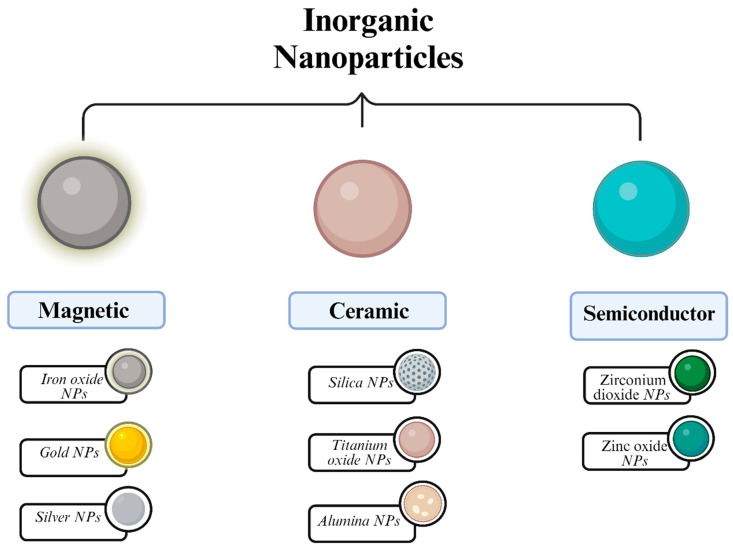
Classification and subtypes of iNPs [15].

Inorganic NPs (iNPs) are composed of inorganic atoms bound with covalent or metallic bonds [13]. They can be synthesized from semiconductors, ceramics, or magnetic metals (Figure 3). The main core of the iNP is formed by the crystallization of inorganic salts, arranged three-dimensionally with bound atoms. As a result of that, the particles are highly organized and orderly positioned, which increases their resistance against destabilization triggers.

The size of the surface is a crucial factor in the iNP’s activity capacity, which is also related to their toxicity potential [14]. Such attributions are the main factor behind the wide-ranged distribution of inorganic NP applications. For example, the type of ligand that is chosen for the NP surface significantly influences its application. Surface ligands can alter, even change the function of NP, thereby affecting the preferred area of application (as greatly exemplified in Section 3) [15].

Since inorganic NPs can be efficiently applied as therapeutic agents, they are one of the most preferred NP types on a commercial scale [16]. These inorganic NPs are extensively produced for mainly anticancer, antioxidant, and antibacterial applications [13]. Depending on the iNP’s structure, certain iNP subclasses are preferred and more efficient as agents.

#### 2.1.1. Magnetic NPs

Magnetic nanoparticles (MNPs) are described as inorganic NPs that show a response to an applied magnetic field [17]. They are generally composed of magnetic materials, either solely or combined, including nickel, iron, cobalt, and their oxides [18]. MNPs are subdivided into two classes, single and multi-domain, based on size differences. Specifically, MNPs typically around 10–20 nanometers are considered among single magnetic domain NPs. 

MNPs possess various properties, including magnetic anisotropy, low Curie temperature, magnetic coercivity, and high heating efficiency [17]. Those properties are primarily influenced by finite-size effects resulting from quantum confinement of the electrons, different crystal structures, and surface effects that are related to symmetry breaking of the crystal structure, at the surface of the particle [19]. Additionally, single-domain MNPs exert superparamagnetic behavior under optimum temperatures [20]. Superparamagnetic behavior can be defined as the change in magnetization of MNPs through thermal energy due to their small volume [21]. MNPs that exhibit superparamagnetic behavior typically have a uniform surface and a size smaller than 20 nm. These MNPs can become permanently magnetized when subjected to an external magnetic field [22]. In other words, in the absence of the electromagnetic field, the net magnetic moment of a system containing MNPs will be zero at high enough temperatures. On the contrary, in the presence of a magnetic field, there will be a net statistical alignment of magnetic moments in MNPs, similar to paramagnetic materials but with a magnetic moment involving multiple atoms up to 10⁴ times larger [23]. 

This property, characterized by the absence of residual magnetization after the external field is removed, enables MNPs to avoid agglomeration and maintain their colloidal stability [21]. Overall, it is considered one of the key factors that make MNPs distinctive for use in biological areas including magnetic resonance imaging (MRI), drug delivery systems, magnetic hyperthermia (MH) treatment, gene delivery, and tissue engineering [24,25,26]. 

MNPs are frequently used in many biological applications. Iron oxide nanoparticles (IONPs), magnetite (Fe_3_O_4_), and its oxidized form maghemite (γ-Fe_2_O_3_) are the most studied MNPs for biological uses due to their ease of functionalization, biodegradability, biocompatibility, and low-cost synthesis [27]. Briefly, IONPs possess significant antibacterial properties and are widely employed in diverse applications, such as drug delivery systems. Further details on these applications will be discussed in subsequent chapters.

#### 2.1.2. Ceramic NPs

Ceramics are materials that have been solidified by the application of pressure and/or heat. They are composed of a blend of metals and nonmetals, including one or more metals mixed with a nonmetal, or numerous nonmetallic components combined with a nonmetal [28]. Applications for ceramic materials include glass, cement, clay minerals, and other materials. Furthermore, a variety of medical applications employ ceramics made of calcium phosphates, silica, alumina, zirconium, iron oxides, carbonates, and titanium dioxide because of their favorable interactions with human tissues. By way of example in the dental industry, metal–ceramic alloys are utilized for crowns, and compounds based on calcium hydroxide and phosphate are employed as endodontic filling materials [29]. 

In general, they resist strong chemicals better than metals and polymers and act as heat and electrical insulators. Mechanically, they are brittle and hard. They retain their great hardness, superior resistance to heat and corrosion, and electrical insulating qualities at the nanoscale [30]. Inorganic substances such as alumina or silica make up the majority of ceramic NPs. All metals, metal oxides, and metal sulfides can be utilized to create nanosystems with different dimensions, shapes, and porosities, so the NP core is not restricted to only these two substances [31]. Because they do not swell or change porosity in response to pH variations, these particles completely shield entrapped molecules, such as proteins, enzymes, and medications, from denaturing as a result of variations in the external pH and temperature [32]. Furthermore, nitric oxide donation and detection using hybrid nanospheres containing cadmium selenide quantum dots have shown excellent selectivity and efficient sensitivity [33]. 

New ceramic materials and NPs are currently being developed quickly for use in biological applications. Advanced synthetic techniques have been employed to enhance the physical–chemical properties of nanoscale ceramics, including hydroxyapatite (HA), silica (SiO_2_), titanium oxide (TiO_2_), and alumina (Al_2_O_3_), to minimize their cytotoxicity in biological systems. One of the most widely used applications of ceramic NPs in biomedicine is controlled drug release. The dose and size are crucial in this discipline. Additionally, the high stability, high load capacity, ease of absorption into hydrophobic and hydrophilic systems, and various modes of administration (oral, inhalation, etc.) of NPs make them a promising tool for controlling drug delivery. Furthermore, a directed action is made possible by a range of organic groups that can be functionalized on its surfaces [34]. Similar to metallic NPs, ceramic NPs have also been widely used in bone tissue engineering because of their many advantages for bone tissues and cells. Because the components of some ceramic NP kinds, notably HA and tricalcium phosphate (TCP) NPs, are similar to those of natural bone, they are ideal as materials to replace bone [35]. 

To sum up, ceramic NPs offer a promising substitute for synthetic counterparts in medication delivery. Stability against pH and temperature changes is their primary benefit. They also form easily into a wide range of shapes, sizes, and forms, which makes them perfect for delivery systems. However, a major obstacle to its practical implementation is the paucity of studies.

#### 2.1.3. Semiconductor NPs

Semiconductors are solid materials that possess a crystalline structure [36]. As their name suggests, semiconductors have conductivity and electron energy gaps that lie between conductors and insulators. Certain semiconductors, such as zirconium dioxide (Zr O_2_) and zinc oxide (ZnO), are commonly used in the synthesis of semiconductor NPs (sNPs) [37]. The application of sNPs is extensively discussed in certain areas, such as catalysis, sensors, optical coatings, and especially ceramics and dentistry [36,38]. 

Zirconium dioxide (ZrO_2_) is a widely preferred semiconductor crystalline material for NP development. ZrO_2_ has gained attention in several research areas due to its biocompatibility, non-toxicity, high compression resistance, and fracture strength [39]. Thanks to its resistant feature, ZrO_2_ is largely involved in dental implants and certain biomaterials to enhance the resistance of the material [40]. These properties have extended to the use of ZrO_2_ and ZrO_2_-based materials in distinct areas such as thermal coating, energy storage, and many biomedical applications, which are very similar to general sNP applications [41]. In biomedical applications, ZrO_2_ NPs can also exhibit antimicrobial activity through charge-based interaction with bacterial cells [42]. 

Another common semiconductor material that is used in NP synthesis is ZnO. It possesses several unique characteristics as a highlighted semiconductor material, including high chemical stability, radiation absorption, and photostability [43]. It has a wide range of application areas, including but not limited to electronics, rubber and textile industries, and pharmaceuticals. ZnO NPs exhibit significant thermal and mechanical stability, along with the high binding and gap energy [44]. Thanks to its rich chemical and physical properties, ZnO is one of the leading materials used in NP synthesis for many applications such as electronics, sensors, and solar cells [45]. In addition, similar to ZrO_2_, ZnO NPs are also included in antimicrobial activity research [46]. A crucial mechanism behind the antimicrobial activity of ZnO NPs was demonstrated in an *in vitro* study, causing bacterial death by the release of hydroxyl radicals [47]. Moreover, ZnO NPs are heavily discussed in the current literature for biomedical application, particularly with green synthesis methods, including drug delivery, anticancer research, and bioimaging [48]. Detailed examples of these materials, along with other types of inorganic NPs, are discussed in the application section.

### 2.2. Carbon-Based NPs

Carbon has a notable place in nanotechnology due to its ability to form long and resistant chains. This unique capability of carbon is constantly utilized in the field of NPs, known as carbon-based NPs. Carbon-based NPs possess significant characteristics such as high chemical stability, powerful heat and electrical conductivity, high optical absorption, and luminescence [49]. Consequently, they are involved in many study fields, including biosensors, drug delivery, cancer, and cellular therapy; *in vivo, in vitro*, and optical imaging; and so on [50]. Additionally, carbon-based NPs can exhibit antibacterial activities by directly interacting with the bacteria and potentially causing oxidative stress that creates membrane damage, leading to cellular death [51]. 

**Figure 4:**
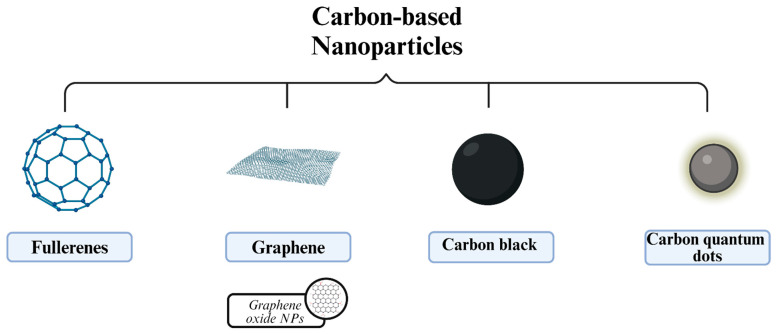
Classification and subtypes of carbon-based NPs [49,51].

Carbon-based NPs come in different forms, including graphene, fullerene, and carbon black NPs (Figure 4). These allotropic forms of carbon are specifically highlighted thanks to their significant chemical and physical properties, which are extremely essential in nanotechnological applications.

#### 2.2.1. Graphene

Graphene is a carbon-based nanostructure that consists of single-layer carbon atoms. It has a unique electrochemical nature thanks to its high thermal conductivity, hydrophobicity, and density [52]. Since it is equipped with a wide range of chemical and physical properties, it is applied in many areas. To add, graphene derivatives, including graphene oxide (GO) and chemically modified graphene, have also received high attention in the field [53]. GO contains high oxygen-containing functional groups that facilitate its functionalization for specific areas [54]. As a result of these, it is usually utilized with an NP, rather than as an NP by itself, as a combined material to form a hybrid structure to add properties of graphene to the structure. This hybrid structure can be formed in two ways: either the NP can be decorated on graphene sheets (forming a composite), or the surface of the NP can be modified with graphene or its derivatives [55].

Significant numbers of inorganic metal NPs have been decorated with graphene to develop a hybrid structure that possesses the combination of both material’s properties. This property is utilized in various areas, such as drug carrying, cell labeling, and photothermal therapy [56]. Combined with the high functionalization opportunity, graphene’s high surface area and molecular composition allow for significant amounts of drug loading, increasing the preference for GO-based materials to a significant level [57]. Furthermore, graphene-based nanomaterials are widely used as a supportive material in biomedical applications, especially in regenerative tissue studies [58]. The biomaterial that aims to be used in the regeneration of the tissue needs to possess certain characteristics, primarily the ability to promote and modulate cellular progress, such as cell growth, proliferation, and signals [59]. Since graphene meets these required characteristics with high biocompatibility, multiple types of graphene structures are currently used in tissue engineering applications as well [60]. In these terms, we have elaborated the application of graphene as an additive material to other types of NPs, rather than its sole NP application.

#### 2.2.2. Fullerenes

Fullerenes are an allotrope of carbon that consists of hollow clusters of sp-2-hybridized carbon atoms, making them one of the primary nanomaterials used extensively [61]. These molecules form spherical cages, with carbon atoms located at the corners of polyhedral structures [62]. Fullerenes have unique chemical reactivity and electronic traits; thus, they have been used in various fields of science as nanosensors [63], antioxidants [64], material in solar cells [65], therapeutics in nanomedicine [66], and so on. Despite the rich characteristics of fullerenes, their general application on an NP basis consists mainly of one type of derivative: C_60_. Fullerene C_60_ is one of the most investigated derivatives, dominantly applied in NP technology compared to other derivatives. C_60_ fullerene is a spherical molecule that has 60 carbon atoms, making up 20 six-membrane and 12 five-membrane rings [67]. The most highlighted feature of C_60_ is its significant radical scavenger activity, as it is called a “radical sponge”, thanks to its large number of double bonds and cage-like structure [68]. On the contrary, C_60_ can also lead to the synthesis of reactive oxygen species (ROS), but under specific conditions. When C_60_ is exposed to visible or UV light, it causes excitation and enables the creation of ROS by the excess energy [69].

In addition, fullerenes are also widely used in dermatology. Thanks to its capability of showing significant antioxidant activity and UV-based damage protection, many fullerene-based patents have been created in dermatological applications, such as in hair care, makeup, deodorants, and so on [70]. In light of this, many products contain fullerene as an ingredient, including but not limited to creams, lotions, soups, and face masks [70]. To add more, fullerene’s molecular structure is suitable for modification and NP synthesis; thus, it is usually preferred for drug delivery systems as well [71]. Thanks to its superior molecular structure, its involvement in delivery systems is widespread, such as in nucleic acid and topical drug delivery, delivery to the central nervous system, and control of antibacterial and oxidative stress [72]. In addition, fullerene is also extensively used in cancer diagnosis and therapy as a carrier, material, and drug, especially due to its light-sensitive ROS generation [73].

#### 2.2.3. Carbon Black NPs

Carbon black NPs (CBNPs) are nanomaterials composed mainly of carbon, along with trace amounts of other elements such as hydrogen and oxygen [74]. They are formed during the thermal decomposition processes of hydrocarbons that are either in a liquid or gaseous state, resulting from the change of temperature caused by lack of oxygen [75]. CBNPs possess excellent electrical conductivity and versatility, and are easily functionalized, in addition to being available and cost-effective [76]. These properties make them one of the most widely used materials in industrial applications. Specifically, CBNPs are mainly used as reinforcing fillers in the rubber industry to enhance the physical properties of rubber [77]. Additionally, they have been used in the design of electrochemical sensors [78], lithium and sodium batteries [79], and as additives to polymers [80]. 

Apart from industrial applications, CBNPs are mainly utilized through hybridization with other types of NPs to enhance their activity and application. For instance, a recent study demonstrated a hybrid platform using carbon black and palladium NP to create an enhanced immune sensor for cancer cells [81]. Human serum samples were also included in the research to further point out the potential of this hybrid NP platform in real-time application. Similarly, another hybridization was performed using gold NPs and carbon black material to develop more sensitive drug detection [82]. In both of these applications, it was stated that the high surface area-to-volume ratio and electrochemical properties with electron transfer advantage were the highlighted features of the CBNPs.

However, despite their widespread use, CBNPs can promote oxidative stress, leading to cellular damage and inflammation, especially in the lung and cardiovascular systems [83]. This toxicity, arising from their high surface area-to-volume ratio, has led to CBNPs being considered a risk factor, highlighting the need for further studies. This is why they have a limited role in biological applications, especially compared to other carbon-based NPs.

#### 2.2.4. Carbon Quantum Dots

Carbon quantum dots (CQDs) are environmentally friendly, cost-effective, low-toxic carbon-based NPs with a size smaller than 10 nm [84]. They are also known as small carbon-based NPs with unique electronic properties, which underlie their fluorescence properties in many areas [85]. Their unique structure makes them promising in various fields such as optronics, biomedicine, drug delivery, sensing, and catalytic applications [86,87,88]. CQDs can also contain diverse functional groups such as amine, carboxyl, carbonyl, hydroxyl, etc. These groups highlight CQDs potential for biological, organic, and polymeric functionalization and create opportunities for their use in diverse areas [89]. 

Furthermore, multiple studies have shown that CQDs are biocompatible and effective for fluorescence imaging, providing an alternative to heavy metal quantum dots for *in vitro* and *in vivo* applications [90]. Additionally, CQDs synthesized using citric acid and branched polyethyleneimine (BPEI) at low temperatures exhibited enhanced fluorescence, increasing their potential for chemical sensing [91]. Similar bioimaging applications were demonstrated using CQD NPs against cells and tumors, highlighting their potential for imaging purposes [92].

Additionally, it was highlighted that CQDs with a positive charge can show considerable antibacterial activity by interacting with negatively charged elements of the bacterial cell wall, causing structural damage and eventually cell death [93]. To specify, the antibacterial effectiveness of positively charged CQDs against Gram-positive and Gram-negative bacteria, *Streptococcus aureus* (*S. aureus*) and *Escherichia coli* (*E. coli*), respectively, was demonstrated [94]. Moreover, numerous studies with resembling results are present in the current literature, which will be elaborated on in the following chapter [95,96]. 

Overall, it can be concluded that the unique characteristics of CQDs, such as biocompatibility, ease of functionalization, affordability, chemical inertness, antibacterial activity, and low toxicity, make them promising candidates for a variety of uses (Table 1).

### 2.3. Organic NPs

Organic NPs can be defined as solid particles made up of natural or synthetic organic molecules, generally ranging in diameter from 10 to 100 nm, but, preferably, they can reach up to 1000 nm within the definition [142].

**Figure 5:**
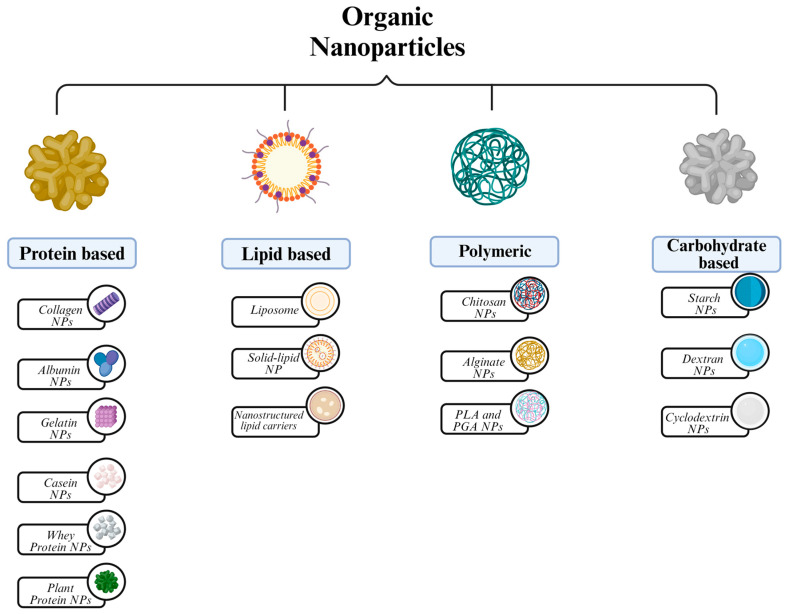
Classification and subtypes of organic NPs [142].

Organic NPs exhibit important characteristics such as non-toxicity and biodegradability, since they are composed of proteins, lipids, polymers, and carbohydrates (Figure 5). These properties, consequently, enable organic NP utilization in various biomedical areas including targeted drug delivery, bioimaging, cancer therapy, and biosensing [143]. Moreover, certain properties such as size, shape, and surface morphology also hold importance in determining the therapeutic effectiveness of organic NPs [12].

Overall, the compatibility and effectiveness of organic NPs in these applications not only underscore their potential but also give rise to promising opportunities for further research and development.

#### 2.3.1. Polymeric NPs

Polymers are composed of repeated monomers that show extended diversity, as their structure can show variables in terms of size, structure, and function [144]. The diversity in the composition and flexible synthesis of polymers has led to a favorable shift towards the application of polymeric NPs (PNPs). Since they are synthesized from natural polymers, making them biocompatible and biodegradable, they possess significantly lower toxicity levels [145]. The low toxicity of polymers is a contributing factor to their preferred usage, especially in drug and gene delivery systems. Given that NP-based delivery systems have significant advantages, such as specificity in targeting, stabilization, and protection of the delivered agent, and flexible release conditions, PNPs are frequently used in drug delivery [146]. 

The polymers used for NPs can be either natural or synthetic. The most common natural polymers used are chitosan and alginate. Among synthetic polymers, poly (lactic acid) (PLA); poly (glycolic acid) (PGA); and the copolymer of PGA and PLA, poly(lactide-co-glycolide) (PLGA), are the most commonly used in PNP application. Each polymer is preferred for different and similar applications based on its unique properties.

##### Chitosan NPs

Chitosan is one of the most abundant biopolymers found in nature, known for its high biodegradability and antimicrobial activity [147]. In addition to possessing properties found in general polymers for NP synthesis, such as high biocompatibility, low toxicity, and modifiability, chitosan can also improve absorption rate, and exhibit mucoadhesive characteristics and antimicrobial activity [112]. 

Chitosan NPs are used in food packaging [148], antioxidant and antibacterial research [149,150], drug delivery [151], and other applications. Among these applications, chitosan NPs are commonly preferred for drug delivery, similar to other types of PNPs, thanks to their cationic structure and presence of amine groups, which are essential for uptake by the cellular membrane [152]. Chitosan’s attributes, especially its mucoadhesive characteristics and modifiability, are also utilized in cancer treatments [153]. 

Many metal ions, such as silver ions, are used with chitosan NPs to generate enhanced effects, especially in antibacterial research. Chitosan NPs can directly disrupt the bacterial cell walls and membranes and can be modified with these metal ions to further increase their activity [154]. Since silver NPs have proven to be effective antibacterial agents [155], they are often combined with chitosan to enhance antibacterial activity [156]. A similar study also showed the antioxidant, antibiofilm, wound healing, and antifungal potential of chitosan with silver NPs [157]. This enhanced antibacterial activity of silver–chitosan NPs is also being explored in food preservation research [113]. 

Along with the utilization of metal ions in chitosan NPs, similar polymers and different bioactive molecules are also used in various areas. For instance, it has been established that adding lysozyme, an enzyme capable of hydrolyzing bacterial cell walls [158], to chitosan NPs significantly improves antibacterial activity [159]. Lysozyme-added chitosan NPs showed increased activity compared to chitosan NPs and solo chitosan application. Chitosan is also commonly combined with alginate in NP synthesis, which will be briefly covered in the next section.

##### Alginate NPs

Alginate is also a significant material in NP applications, due to its tendency to be functionalized by its free carboxyl and hydroxyl groups. Certain characteristics, such as hydrophobicity, solubility, and specificity, can be modified through oxidation, esterification, sulfation, and amidation reactions [160,161]. Like the other PNPs, alginate is common in drug delivery systems. Much research has demonstrated the efficient drug delivery capabilities of alginate NPs [162,163]. Due to its high biodegradability, biocompatibility, potential for modification, and pH sensitivity, alginate is a very effective material for delivering various biomolecules. This effectiveness has led to its approval by the FDA for use in drug delivery systems [118]. Like chitosan, alginate also exhibits mucoadhesive properties, thanks to its ability to form weak bonds with mucin [164]. All of these attributes of alginate (NPs) are utilized in the transportation of various bioactive molecules, such as CRISPR plasmid DNA [165], the bioactive ingredient curcumin [166], peptide hormone insulin [167], and so on.

Alginate is often combined with chitosan in the application of PNPs. For example, the combination of alginate and chitosan NPs shows significant potential in delivering drugs and natural compounds [168,169] to various regions, including the intestine [170]. Besides drug delivery, this hybrid NP is also studied and applied as a nanofertilizer [171], vaccine carrier [172], antibacterial agent carrier [173], and so on. In fact, alginate is used not only as a general NP in drug delivery applications but also in the production of nanogels [119]. Nanogels offer significant advantages, such as a reduced potential to cause adverse effects, an increased capability to carry the desired biomolecule, and controlled release of these biomolecules [174]. Additionally, they exhibit unique sensitivity to important factors such as temperature and pH, which are essential in drug release [175]. In this regard, many researchers have demonstrated the synthesis and application of alginate nanogels, particularly in biomolecule transport and release [128,176,177].

##### Polylactic Acid and Polyglycolic Acid NPs

PLA is a biodegradable polymer material that is produced from renewable sources such as wheat, corn, and starch [178]. Alongside its high biodegradability, it also possesses significant biocompatibility, mechanical strength, and composability [179]. Moreover, it requires considerably lower energy for production and can be degraded to carbon dioxide (CO_2_) and biomass [180], which is a great advantage in biomedical applications. Additionally, several characteristics of PLA have been discussed as advantageous in packaging and fiber application [181]. Similar to chitosan and alginate, PLA NPs are also combined with other types of polymers. For example, a study used chitosan-modified PLA NPs to increase the bioavailability of antitumor agent ursolic acid [182]. As discussed above, chitosan was the preferred polymer in this combination due to its mucoadhesive characteristics. Similarly, PLA NPs were combined with polyethylenimine to increase the antimicrobial activity of carvacrol in food preservation [183]. 

PGA is another preferred type of polymer used in both polymer-based and NP applications. Its application is as widespread as that of PLA. Even though PGA and PLA have similar structures, both possess unique properties: high mechanical properties, thermal stability, and faster degradation for PGA, and high transparency and rigidity for PLA [181]. In current research, PGA is utilized in NP applications as a part of a copolymer rather than a primary material. The main copolymer of PGA that is widely used is polylactide-co-glycolide (PLGA), an FDA-approved biodegradable copolymer. Like the other types of polymers that have been discussed, PLGA shows significant biodegradability and biocompatibility, which is a crucial factor for its use in delivery systems [184]. Additional characteristics of PLGA include capacity for surface functionalization, CO_2_ as an end product after the degradation, and involvement of wide-ranged biomedical applications [120]. Advantages and additional characteristics of PLGA include increased protection on the biomolecule that has been loaded, reduced dose frequency and degradation time, and maintains sustained drug release [185]. This is why PLGA is one of the safest, most preferred, and most effective polymers utilized in PNP applications, which will be covered extensively in the NP application section.

#### 2.3.2. Lipid-Based NPs

Lipid-based nanoparticles (LBNPs) are a versatile class of NPs that are widely used in current medical studies, particularly as effective drug carriers. They possess various advantages such as non-toxicity, biocompatibility, and biodegradability when compared to other classes of NPs [186]. Due to their non-toxic nature, they are utilized for the transportation of both hydrophilic and hydrophobic molecules, as well as for controlling the release of drugs and enhancing their efficiency [129]. Additionally, one advantage of LBNPs is that they can be functionalized to include antibacterial agents, which are essential for improving wound healing, promoting skin regeneration, and reducing inflammation [187]. This capability, consequently, not only underlines the importance of LBNPs but also creates substantial opportunities to develop innovative applications in the field of NPs. 

In the current literature, LBNPs are generally categorized into three groups: liposomes, solid lipid NPs (SLNs), and nanostructured lipid carriers (NLCs). 

##### Liposomes

Liposomes are artificially produced spherical nanomaterials that are widely employed in medical [188], cosmetic [127], and industrial fields [189] due to their distinct characteristics, such as biocompatibility, stability, and biodegradability. Along with their long-standing application, they are referred to as the earliest generation of lipid NPs [190]. Currently, they are often utilized as delivery systems, particularly for targeted drug delivery, as they can efficiently encapsulate both hydrophilic and lipophilic drugs in the aqueous phase or bilayer membrane, respectively [191].

The structure of the liposomes consists of one or more lipid bilayers, usually composed of phospholipids such as phosphatidic acid, phosphatidylglycerol, phosphatidylserine, phosphatidylcholine, and phosphatidylethanolamine [192]. The composition of these components can significantly affect the properties of liposomes, including size, fluidity, rigidity, stability, and electrical charge [193]. 

Moreover, liposomes can be subdivided into four groups, small unilamellar vesicles, large unilamellar vesicles, multilamellar vesicles, and multivesicular vesicles, based on their size and the number of bilayers that they contain [194]. Additionally, it is important to mention that both the number and size of the bilayers significantly affect the amount of drugs that can be encapsulated [195]. Liposomes can also contain cholesterol in addition to phospholipids. Cholesterol plays an important role in liposome content by improving stability and fluidity, reducing lipid exchange, and enhancing structural rigidity [196]. Alternatively, polymers can be incorporated with liposomes since they are suitable for surface modification due to their flexible bilayer structure. For example, PEGylation is currently considered an effective method to increase stability and extend the circulation time of liposomes *in vivo* [197]. In addition, several studies in the literature regard liposomes as promising antibacterial agents due to their ability to deliver antibiotics directly to the site of infection, effectively targeting and eliminating resistant bacterial strains [198].

In summary, liposomes are considered essential in medical, pharmaceutical, cosmetic, and industrial areas due to their unique characteristics including the ability to encapsulate various substances, targeted delivery potential, high drug-loading efficiency, and controlled release function. This versatility allows for extensive use in diverse applications, such as cancer therapy [199], gene delivery [200], vaccine formulation [201], and skin care treatments [202], highlighting their broad utility in various areas. 

##### Solid Lipid NPs

SLNs are the first generation of LBNPs, containing a solid lipid core matrix stabilized by surfactants or polymers [203]. They are spherical, ranging in size between 50 to 1000 nm, and are especially known for being efficient colloidal delivery agents [204]. SLNs also possess favorable characteristics such as physical stability, protection of labile drugs, delivery of both hydrophilic and lipophilic molecules, ease of production, and low cost [205]. These properties ultimately make them valuable materials in applications such as drug delivery [206], cancer therapy [207], and cosmetics [208], similar to liposomes. 

Even though SLNs are considered alternatives to conventional carriers such as liposomes or polymeric NPs, it is important to consider certain drawbacks associated with SLNs, such as low drug loading efficiency, limited space for encapsulation, and the possibility of drug expulsion resulting from their desired, tightly packed, crystalline structure [205]. However, considering both advantages and disadvantages, SLNs still hold great importance in covering a significant portion of the LBNPs, in terms of application and preference for further studies. 

##### Nanostructured Lipid Carriers

NLCs, generally referred to as the second generation of LBNPs, are designed to overcome the limitations of SLNs. They are defined as colloidal drug delivery systems characterized by containing both solid and liquid lipids in their core matrix [209]. They exhibit improved loading efficiency and higher loading capacity as a result of their imperfect crystalline structure when compared to SLNs [210]. Furthermore, NLCs are capable of avoiding drug expulsion as they incorporate liquid lipids, which consequently enables them to prevent crystallization [186]. 

Overall, NLCs are widely employed nanocarriers in targeted drug delivery [132], cancer therapy [211], the food industry [133], and cosmetic applications [212], as well as other types of LBNPs. Since they possess numerous advantageous features, NLCs are considered important for future applications and are likely to be crucial in advancing NP technologies.

#### 2.3.3. Carbohydrate NPs

Carbohydrate-based chemicals or carbs themselves make up the majority of the nanoscale structures known as carbohydrate NPs. They are frequently referred to as carbohydrate-based NPs or glyconanoparticles. These NPs’ biocompatibility, biodegradability, and functional flexibility have sparked a lot of attention [137].

##### Starch NPs

A natural source of stored energy, starch is a polymer that is renewable, biodegradable, and generated by a wide variety of plants. Starch molecules are composed of anhydrous glucose units, which are typically gathered in distinct, independent granules. Starch, synthesized in dense granules ranging from 1 to 100 μm, consists mostly of amylopectin (~70–75%) and amylose (~25–30%) [213]. The packing of amylopectin double helices, made up of two polyglycoside chains, in a unit cell determines the degree of crystallinity in starch, which can vary significantly (14–45%) based on the starch source [214]. In nature, it is the second most abundant biomass material. Despite years of research, a widely recognized model for starch structure does not exist due to its intricacy [215]. Starch is typically classified into four main categories: cereal, tuber, legume, and other starches. More research is being conducted on starches taken from diverse genetic and botanical sources. In addition, starches can occasionally be chemically and physically altered to suit certain purposes [216]. Starch impacts various food properties, including moisture content, consistency, appearance, and shelf life [217]. It is frequently used to make custard, sauces, pie fillings, soups, gravies, and stews. In addition to acting as a thickening or bulking ingredient and enhancing the texture of a meal, it also serves as an adhesive, gel, and other agents [218]. 

Starch has the ability to create bio-NPs, often referred to as starch NPs. These starch NPs, unlike natural starch, are characterized by their smaller size, typically ranging from tens to a few hundred nanometers by exhibiting a spherical form [219]. Furthermore, starch NPs possess distinct qualities such as a large surface area per unit mass, low diffusion limitation, better absorption capacity, higher biological penetration rate, and greater solubility compared to natural starch granules [220,221]. They are also non-toxic and suitable for the environment [222]. The use of starch NPs as a filler in composites coincided with the growing interest in nanomaterials. Researchers [223,224] discovered that the addition of starch NPs enhanced the mechanical and biodegradability properties of the composites. Furthermore, it has been claimed that the starch NPs are applicable in various domains, including pharmaceuticals, cosmetics, and foods [225,226]. 

##### Dextran NPs

Dextran is a neutral bacterial exopolysaccharide that is both extremely biocompatible and biodegradable. Its straightforward but distinctive biopolymeric composition makes it ideal for use as a nanodrug carrier, nanomedicine, cell imaging system, and nano-biosensor. The fact that it is highly water-soluble and exhibits minimal cellular toxicity after drug delivery is crucial. Renal failure is unlikely because the body is capable of fully metabolizing dextran. NPs based on dextran exhibit enhanced solubility in water, a large cargo capacity, inherent viscosity, and short storage duration [227]. Despite being nothing more than an assembly of glucose molecules, dextran finds widespread application in medicine, primarily as an adjuvant that lowers blood viscosity and inhibits the development of blood clots [228]. Additionally, dextran has been used in nanomedicine, a relatively new field that uses submicron particles for both diagnostic and therapeutic applications. To circumvent NP and opsonin interactions, dextran is utilized as a substitute for PEGylation [229]. Drugs that are administered orally (such as ibuprofen) have improved qualities when dextran is added to SLNs [230]. Additionally, dextran was employed to preserve and stabilize distinct protein structures of insulin, hemoglobin, streptokinase, albumin, and asparagines [231,232]. By reducing immunogenicity and lengthening the duration of protein biodistribution, dextran supplementation maintains the high activity of proteins. Given that dextran is widely used, can efficiently metabolize, and is cleared by the liver and spleen, it is tempting to employ dextran as the foundation of a nanosystem as well as an additional material [233].

##### Cyclodextrin NPs

Cyclodextrins (CDs) are starch-digesting enzyme-derived cyclic oligosaccharides. Primary and secondary hydroxyl groups of the α, β, and γ CDs are situated on the narrower and wider rims of a truncated cone-shaped structure, respectively, and contain six, seven, and eight glucopyranose units. In addition to these conventional forms, large-ring CDs (LR-CDs) are also available. Being composed of more than eight glucopyranose units, LR-CDs possess bigger cavities. This structure enables LR-CDs to accommodate larger guest molecules, enhancing their applicability in forming inclusion complexes. [234]. The toroidal shape and non-polar interior of CDs give them a remarkable ability to form supramolecular host–guest interactions [235]. Since CD molecules have unique architectural characteristics, allowing for them to form inclusion complexes with a variety of molecules, including ions, proteins, and oligonucleotides, they offer notable advantages [236]. 

The literature has extensively documented the several medical applications of CDs due to their low toxicity and immunogenicity. Increasing the solubility of hydrophobic medications in water is crucial. To improve the CD derivatives’ characteristics, particularly their pharmacological action, chemical modifications have been made to them [237]. 

Pharmaceutical researchers have significantly used CDs to improve the bioavailability, aqueous solubility, and stability of a number of medicinal medicines due to their remarkable capacity to trap a guest molecule inside of their hydrophobic cavity [238]. Recently, CDs have been incorporated into polymer systems to create nanocarriers that are particularly helpful for increasing the solubility of hydrophobic medications. Compared to microparticles, these CD NPs have a larger surface area, and they are generally more stable than liposomes. With exhibiting significantly less toxicity, more surface area than microparticles, and higher stability than liposomes, CD-conjugated NPs show diverse benefits, including increased drug solubility, improved encapsulation efficiency, and efficient drug loading. They also act as drug carriers to a particular target site, such as cancer cells [239]. After systemic delivery, CD-based NPs can enhance bioavailability, alter drug metabolism, lessen toxicity, and lengthen the biological half-life of medications [240,241].

##### Protein NPs

Usually, naturally found or synthetically synthesized proteins are used to create protein NPs. A family of natural molecules known as proteins have special functions and applications in the material and biological sciences. Because of their amphiphilicity, which enables them to interact favorably with both the drug and solvent, they are thought to be the perfect materials for taking NPs. Biodegradable and metabolizable, natural protein-derived NPs can have their surfaces modified to facilitate the attachment of medicines and targeted ligands [242]. They have successfully included both water-soluble (e.g., human and bovine serum albumin) and insoluble (e.g., gliadin and zein) proteins from a range of proteins [242,243]. 

Enzymes found within the human body have the ability to destroy protein-based nanocarriers. Furthermore, research has shown that protein NPs only slightly or barely cause an immunological response [244]. Proteases also interact with hydrophilic and hydrophobic medications and solvents because of their amphiphilic nature. Since they include a lot of hydroxyls, amino, and carboxyl groups, they can be chemically modified. Consequently, one or more ligands and therapeutic molecules can be covalently or noncovalently bound to protein NPs. This provides a great deal of surface customization potential [245,246].

##### Collagen NPs

By being the most prevalent protein in the mammalian body, collagen is a structural protein in vertebrates that makes up 20–30% of all proteins in the body. Covalent crosslinks between tropocollagen molecules finally form to make collagen. This protein has broad applications in medicine because of its high biocompatibility, low immunological stimulation, and biodegradability [247]. Collagen demonstrates hemostatic qualities, moderate immunogenicity, bioactive material synergism, and good *in vivo* absorption [248]. Biomaterials of distinct physical forms, such as shields, films, sponges, hydrogels, microspheres, sheets, coatings, liposomes, disks, nanofibers, tablets, pellets, and NPs, are produced as a result of techniques including crosslinking, grafting polymerization, blending, and covalent conjugation. These techniques are used to overcome constraints such as enzymatic degradation, weak mechanical strength, and low thermal stability [249]. 

Collagen is an effective delivery system for a variety of substances, including growth hormones, medications, proteins, and DNA. Because collagen is so versatile, it may be altered to create materials with a wide range of durability, structures, and forms. Moreover, collagen can combine with other physiologically active and therapeutic molecules to form complexes. This protein utilizes in a variety of applications, including drug delivery through the creation of microspheres and microneedle [250] formulation of NPs for gene delivery, protein delivery through pellets and tablets [251], gel formation and combination with liposomes for sustained drug delivery [252], cancer treatment [253], and collagen shields in ophthalmology [254].

Collagen NPs have several advantages over other natural and synthetic polymeric NPs, including favorable biocompatibility and biodegradability, low antigenicity, high contact surface, decreased toxicity, and high cationic charge density potential due to their multiple amino groups; however, their ability to cross the blood–brain barrier is limited if their surface is not modified with target compounds. They are small in size, have a lot of surface area, are capable of being absorbed, and can diffuse into water to create colloidal solutions. Moreover, collagen NPs enhance cell retention, are readily sterilizable, and lessen the effects of harmful byproducts produced during decomposition [255]. Due to their compact size, extensive contact surface area, high absorption capacity, and ability to disperse in water to form a stable and clear colloidal solution, collagen NPs have been utilized as drug carriers for the prolonged release of steroids and antibacterial medications, particularly in the field of dermatology [256,257]. Due to its important role in the development of organs and tissues, collagen is frequently utilized in medicine for purposes such as medication administration and tissue regeneration. Numerous cellular processes are impacted by collagen [248].

##### Albumin NPs

Albumin is becoming a more effective protein carrier for peptide or protein-based medicines, both for drug targeting and enhancing their pharmacokinetic characteristics. With a molecular weight of 66.5 kDa, albumin is the most prevalent plasma protein, present in human serum at 35–50 g/L. Albumin, like the majority of plasma proteins, is made in the liver at a rate of about 0.7 mg/h for each gram of liver, or 10–15 g per day; the average half-life of human serum albumin (HSA) is 19 days [258,259]. Acidic and very soluble, albumin is a very strong protein that remains stable in the pH range of 4–9. It can be dissolved in 40% ethanol and heated to 60 °C for up to 10 h without experiencing any negative effects. Its favorable absorption in tumor and inflammatory tissue, readily available nature, biodegradability, and absence of toxicity or immunogenicity combine to make it a perfect option for drug delivery [260].

Because albumin has several distinct drug-binding sites, a substantial amount of medication can be absorbed into the particle matrix, making albumin-based NP carrier systems an appealing approach [261]. Owing to the well-defined main structure of albumin and its high lysine content, albumin-based NPs may facilitate the electrostatic adsorption of positively charged (such as ganciclovir) or negatively charged (such as oligonucleotide) molecules without the need for additional substances [262,263]. Additionally, coacervation, controlled desolvation, or emulsion creation are simple methods for creating albumin NPs in soft circumstances. They are smaller (between 50 and 300 nm) than microparticles and generally have more controlled release characteristics than liposomes, which could increase patient acceptance and adherence [264].

Albumin-based NPs provide a number of unique benefits, including biodegradability, reproducibility, and ease of preparation. Owing to the high protein binding of different medications, these substances can be effectively incorporated into the albumin NP matrix. Because the surfaces of the NPs have functional groups (such as amino and carboxyl groups), covalent derivatization of albumin NPs with drug-targeting ligands is feasible [265]. Additionally, clinical investigations using approved HSA-based particle formulations, such as AbraxaneTM and AlbunexTM [266], support the expectation that they will be well tolerated [267]. Additionally, preparations of protein NPs, particularly HSA, seem to be a good agent for gene therapy since they may prevent serum interactions that are frequently experienced following the intravenous injection of transfection complexes [268,269].

On the other hand, the albumin-bound nanocarrier system (~130 nm) is a noteworthy protein-based nanocarrier used in cancer therapy. According to studies, albumin builds up in solid tumors, which makes it a good candidate for targeted drug delivery. Its therapeutic viability is demonstrated by the FDA-approved albumin-bound paclitaxel (Abraxane) for metastatic breast cancer. Current clinical trials make use of this approach as well [270]. It is made by combining medications such as paclitaxel with HSA in an aqueous solution, then forming drug-loaded albumin NPs (100–200 nm) using high-pressure homogenization [260]. The body’s natural transporter, albumin, uses caveolae-mediated transcytosis to help molecules move across endothelium membranes [271].

##### Gelatin NPs

One proteinaceous substance that can be utilized to make NPs is gelatin. As one of the most extensively utilized animal proteins, gelatin is produced by carefully regulating the hydrolysis of collagen, a key molecule found in skin, bones, and connective tissues [272]. The FDA has accepted gelatin as a polymer, and it is categorized as “generally recognized as safe” (GRAS). Due to its well-established safety record, it is used as a dietary supplement and as an intravenous expander for plasma, under the brand names Gelafundin and Gelafusal [273].

Numerous techniques, including precipitation, phase separation, emulsion–solvent evaporation, microemulsion, and the self-assembly of gelatin molecules or gelatin–drug complexes, can be used to create gelatinous NPs [274]. Numerous hydrophilic and hydrophobic pharmaceuticals, including those for the treatment of cancer, AIDS, malaria, analgesics, infections, muscle relaxants, and inflammation, have been administered by the use of gelatin NPs. Diabetes is treated with topical ophthalmic medications, protein synthesis inhibitors, tissue plasminogen activators, gene delivery, protein medication administration, and vaccine delivery [275,276].

The authors Lu et al. synthesized 600–1000 nm sized gelatin NPs loaded with paclitaxel, which were demonstrated to be effective in identifying and eliminating human RT4 bladder transitional carcinoma cells [277]. It is simple to conjugate gelatin with artificial polymers such as PEG. PEGylated gelatin NPs enhanced the hydrophobic encapsulated compounds’ plasma half-life and anti-inflammatory properties [278]. Additionally, chloroquine phosphate, an antimalarial medication, was encapsulated in these NPs to lessen the adverse effects of systemic exposure [279].

##### Milk Protein NPs

Naturally occurring biologically active chemicals are transported by milk proteins. They fall into two groups according to their structure [280,281]. Caseins and whey proteins (whey) are among the proteins in this group that have spherical and linear structures, respectively [282].

##### Casein NPs

Casein (Cas), the primary protein found in milk, offers several advantages as a drug-carrying NP, including low cost, easy accessibility, and high stability [283]. Cas is made up of four peptides that vary in terms of amino acid, phosphorus, and carbohydrate content but are identical in terms of their amphiphilic nature. Block distribution in the protein chain is demonstrated by casein’s hydrophilic and hydrophobic sections. Phosphorylation of casein peptides results in a negative charge on their surface, which causes them to bind amorphous calcium phosphate nanoclusters. These characteristics allow for casein molecules to aggregate into spherical micelles under the right circumstances. Since Cas makes up 2.75 percent of milk, it is one of the most widely consumed proteins. Innovative research methods have expanded our knowledge of its characteristics and made new uses possible [284].

Cas has a variety of structural and physicochemical characteristics that make using them as carriers of medicines feasible. A few of these characteristics are its significant stability and surface activity, possessing a high capacity to bind to a wide range of ions and molecules, showing remarkable self-assembly and emulsification qualities, and having the ability to bind water and produce gels. CAS proteins may self-assemble into stable micelles in aqueous solutions because they contain clearly defined hydrophobic and hydrophilic regions [285]. Processes involved in nanoencapsulation benefit from CAS’s strong association property. It is becoming known that CAS-based NPs could be used to deliver pharmaceuticals and nutraceuticals [286,287]. Of all the milk proteins, it has excelled in the field of nanomedicine by increasing the bioavailability of insoluble medicines with reduced toxicity, whereas other polymers may have adverse consequences due to buildup in tissues or cells [288]. Cas in particular is a viable candidate for oral medication administration since it can shield the esophagus and buccal epithelia from drug toxicity [289]. With an obvious amphiphilic structure, Cas is a di-block copolymer that may combine with other molecules to produce nanoscale core–shell nanocomposites in an aqueous media. Hydrophobic blocks form the core of the nanocomposite, while hydrophilic blocks build the shell [290]. Owing to its physicochemical and structural characteristics, Cas has been thoroughly investigated for use in pharmaceutics to improve bioavailability. Cas has been shown in earlier research to be a useful nanovehicle for the oral administration of drugs that are poorly soluble in water, including ibuprofen, folic acid, paclitaxel, and docosahexaenoic acid [291,292].

##### Whey Protein-Based NPs

A particular kind of whey protein that has been modified at the nanoscale to improve its functional characteristics comes in the form of whey protein NPs. These NPs are produced by a number of methods, including emulsification, desolvation, and heat treatment. To stabilize the structure, the NPs are then crosslinked using substances like transglutaminase or glutaraldehyde [293].

A member of the transferrin (Tf) family of cationic iron-binding glycoproteins in mammals is lactoferrin (Lf) [294]. It is released in most mammalian external fluids, including tears and saliva, and has an extremely high affinity for binding iron, as observed in milk. Numerous functions of Lf have been demonstrated by research, including iron absorption, antibacterial, antifungal, antiviral [295], antioxidant, and antitumor properties [296]. These health-promoting qualities have led to the widespread usage of Lf in a variety of disciplines, such as dietary and medicinal applications [297,298]. 

By encapsulating bioactive substances, Lf-NPs can be used in food products to improve their nutritional qualities and grant new functional characteristics. Therefore, it is becoming more and more important to evaluate how these systems function when ingested by people. It is possible to do this assessment using *in vitro* digestion models because they are less complicated, more affordable, easier to use, and do not raise any ethical concerns compared to *in vivo* models [299].

In the fields of food science, medicine, and nutraceuticals, Lf NPs are becoming more useful than ever as carriers. These NPs are prized for their capacity to encapsulate and shield bioactive substances, as well as their biocompatibility and antibacterial qualities. They can be created by emulsification, self-assembly, and desolvation, among other techniques [300].

The two primary proteins in whey that have been researched to develop medication nanocarriers are beta-lactoglobulin (β-lg) and alpha-lactalbumin (α-lac). These proteins have several qualities, one of which is their strong resistance to stomach enzymes that degrade proteins [301]. Because of their biocompatibility and capacity to contain bioactive chemicals, β-lg and α-lac NPs hold great potential in the fields of food science, medicines, and nutraceuticals. Under some circumstances, β-lg self-assembles to improve nutrient bioavailability and offer controlled release in functional foods. In the pharmaceutical industry, they enable targeted distribution and enhance the solubility and stability of hydrophobic medications. High encapsulation efficiency and natural origin represent the benefits; however, issues with stability, scalability, and allergenicity still exist. Research is still being conducted to emphasize their potential in a variety of industries by improving stability, minimizing allergenicity, and optimizing preparation [302]. On the other hand, crosslinking agents, heat treatment, and self-assembly are among the methods used for producing α-lac NPs. α-lac NPs are ideal for functional foods in the food industry because they improve nutrient bioavailability and offer controlled release. When properly altered, they can potentially enable the targeted administration of medications by enhancing their solubility, stability, and bioavailability [303].

##### Plant Protein-Based NPs

This novel strategy for medicine delivery makes use of plant protein nanocarriers. Plant proteins, such as gliadin and zein, have a longer drug release ability than animal protein nanocarriers because they are hydrophobic [304,305]. Compared to animal proteins, vegetable proteins are far more affordable and more commonly available. Additionally, there is no chance that they could expose people to animal illnesses, including bovine insanity [306]. 

Some common plant proteins employed in the development of NPs are zein from corn, gliadin, and glutenin from wheat gluten and soy proteins. Proteins found in plant extracts and lesser-known legumes like peanuts and chickpeas are also thought to be sources of NPs. Plant proteins are preferred for creating nanomaterials because they are less immunogenic than animal proteins [307,308].

Proteins make up the majority of the complex that is wheat gluten, along with carbs. Among these proteins are gliadin and glutenin. These proteins are isolated and detected using 70% alcohol. Glutenin has a molecular weight of 106 kDa and is an alcohol-insoluble protein. Gliadin is a class of proteins with a molecular weight of 25–100 kDa that are 70% isolated from alcohol by gluten [309]. Numerous investigations have demonstrated the efficacy of gliadin NPs as drug release controllers for hydrophobic and amphiphilic substances, including amoxicillin, vitamin A, and vitamin E. Gliadin’s structure includes hydrophobic amino acids and glutamine, which allows for it to interact hydrophobically with the cell membrane while also forming a lot of hydrogen bonds with the mucosa’s mucous layer. Because of this, gliadin NPs have demonstrated excellent promise in the creation of oral formulations, particularly for the management of gastrointestinal disorders such as gastric ulcers [310].

A broad class of glycoproteins, or proteins with the ability to bind to carbohydrates, are called lectins. One of the most well-known and intriguing plant lectins is wheat germ agglutinin (WGA). This protein can increase the absorption of oral medication formulations thanks to its high stability, low toxicity, immunogenicity, resistance to proteolytic degradation, specificity in identification, and binding site to glycosylated components of intestinal mucosa [311,312]. In order to develop targeted drug delivery systems and cover a variety of NP types with lectins, numerous studies have been carried out in this sector. Moreover, lectins are helpful in the creation of oral vaccinations. The immunological response of the oral vaccine is improved by NPs coated with lectins and bearing pathogenic antigens that are directed towards the surface of Peyer’s patches in the intestine [309].

Because of the nutritional and functional qualities of legume proteins, which are found in legumes (family Fabaceae or Leguminosae), they are widely used in food products all over the world. Studies on the use of native and modified legume proteins as encapsulating materials for a variety of bioactives are currently being conducted in large numbers. Grass PPI, chickpea protein isolate, pea protein isolate, pea protein isolate, and soybean protein isolate (SPI) are among the legume proteins that are frequently used to create nanocarriers. Nanocomplexes, nanoemulsions, nanogels, and nanofibers are among the resultant nanocarriers [313]. One of the most plentiful sources of plant protein at the moment is soybean. SPI is the fortified form of soy protein. Because soy protein extract contains a balanced mix of polar, non-polar, and pregnant amino acids, it can be used in a wide range of medications. Partial enzymatic hydrolysis has been used recently to treat SPI. The amphiphilic hydrolysates were subsequently self-assembled into NPs to carry curcumin [314]. Another well-liked legume protein for making nanocarriers is pea protein. In particular, PPI—which is distinct from pea grains—has just lately gained the same prominence in academic and industrial research as SPI [315,316].

Typically, peanut protein is one of the top three plant protein sources worldwide. The most widely commercially available form of peanut protein is peanut protein isolate (PEPI), which is made out of a balanced mix of amino acids [317]. Additionally, after modification, PEPI exhibits good surface-active characteristics and is soluble in water. In order to produce NPs through robust intraparticle disulfide bonds, PEPI has recently been denatured and aggregated using heat treatment in conjunction with divalent ions. There is potential for these protein NPs to function as efficient Pickering stabilizers [318].

Curcumin’s anticancer efficacy against several cancer cell lines was markedly increased by a rapeseed protein nanogel loaded with curcumin, which had a high encapsulation efficiency (95%) [319]. The byproducts of the production of sesame oil, sesame proteins, have significant biological significance. They have a 65–80% globulin fraction with potent emulsifying qualities. Sesame proteins were employed as a natural substitute surfactant in this context to stabilize a nanoemulsion containing fish oil that was high in ω-3 PUFA [320]. The 89.7 nm sized optimized nanoemulsions extended the fish oil’s storage life by eight weeks. Moreover, probiotics were spray-dried and encapsulated using soybean protein isolate as the wall material [321].

One creative technique to make the best use of plant leftovers is to use green leaf protein as a nanocarrier in food and medicine products. The larger balanced fraction of necessary amino acids in a product indicates the presence of certain proteins [322]. Because of their superior nutritional profile over cereals, pseudocereals like quinoa, amaranth, buckwheat, and chia have become more significant in the food market. Quinoa proteins have been utilized recently to encapsulate various bioactives, including luteolin, resveratrol, betanin, quercetin, and curcumin [323]. Liu et al. (2022) found that encasing quercetin into quinoa protein nanomicelles improved its bioavailability, water solubility, and stability [324]. The manufacturing of potato starch, a widely accessible and reasonably priced raw food source, yields a byproduct with added value that is called potato protein. Vegetarians and vegans can eat this protein [325]. Potato protein is also extremely amphiphilic and water-soluble, which makes it an excellent choice for encasing hydrophobic substances. For instance, potato protein NPs contained astaxanthin, a xanthophyll pigment. The findings showed that astaxanthin was shielded from digestion-related breakdown by encapsulation. The GIT fluids’ bioaccessibility, bioavailability, and solubility were greatly enhanced [326].

## 3. Applications of Nanoparticles Based on Their Classification

**Figure 6:**
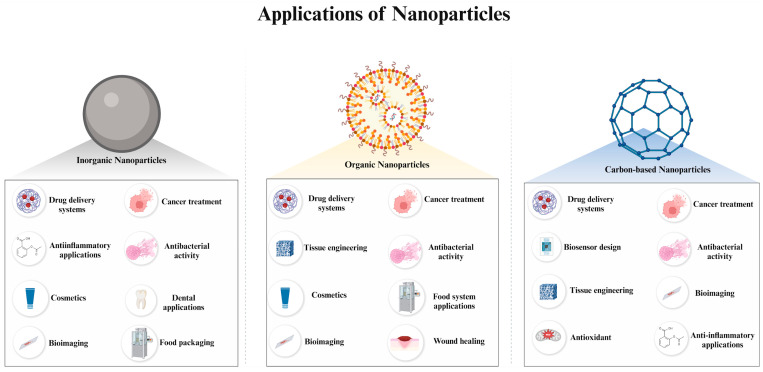
Applications of nanoparticles [327,328].

NPs are composed of various materials, including metals, metal oxides, ceramics, polymers, lipids, carbon-based substances, and proteins. These materials significantly influence the characteristics of NPs, which, in turn, affect their areas of application. In this section, application areas of NPs are examined, specifically focusing on those classified as inorganic, organic, and carbon-based, and how their composition correlates with their field of use is evaluated (Figure 6). 

### 3.1. Inorganic NP Applications

As inorganic NPs are known for their significant potential as therapeutic agents, they are mostly included in antibacterial studies, along with drug delivery systems and anticancer treatments.

#### 3.1.1. Antibacterial Application of Inorganic Nanoparticles

Inorganic NPs constitute a significant portion of NP-based antibacterial research and have been extensively studied and confirmed for their efficiency in numerous antibacterial studies in the current literature. Most subclasses of inorganic NPs can penetrate cell membranes and disrupt intracellular metabolism in many types of bacteria, including drug-resistant strains [329]. It would be challenging to cover all the subtypes of inorganic NPs’ antibacterial activity and applications comprehensively. Therefore, we have included a few major examples in this section and extended their involvement in the area in Table 2.

Inorganic NPs have been extensively studied and confirmed for their efficiency in numerous antibacterial studies in the current literature. Silver NPs are among the most commonly employed inorganic NPs in the biomedical field due to their desirable characteristics, such as antibacterial activity [330]. Various studies in the current literature delve into the antibacterial mechanisms of silver NPs in detail [331,332]. One mechanism involves silver NPs interacting with and modifying the outer bacterial membrane, penetrating and disrupting the membrane’s permeability, ultimately inhibiting bacterial proliferation. To exemplify, a study examining this mechanism showed that silver NPs are excellent antibacterial agents against *S. aureus* and *E. coli*, which cause cellular disturbance and eventually cell death [333]. Another mechanism suggests that silver NPs penetrate the cell membrane and bind to phosphorus or sulfur groups in molecules such as proteins or DNA, consequently altering their configuration and function. This interaction also includes the binding of silver NPs to microbial DNA, inhibiting bacterial replication [334]. 

Moreover, a different mechanism involves silver NPs releasing silver ions that interact with cellular structures and disrupt metabolic processes, causing extensive disruption in bacterial cells. Specifically, after free silver ions are absorbed, they deactivate respiratory enzymes, leading to the generation of ROS. This increase in ROS levels primarily causes the breakdown of the cell membrane and DNA modifications [335].

Furthermore, there are multiple studies in the current literature underscoring the antibacterial activity of silver NPs. To exemplify, a study examined the antibacterial activity of silver NPs on *E. coli* ATCC 8739 [336]. Results revealed that silver NPs, at a concentration of 10 μg/mL, could inhibit the growth of 10⁷ cfu/mL *E. coli* cells. Furthermore, detailed microscopic analyses using SEM and TEM revealed severe damage to the bacterial cells, including the formation of pits and gaps on the cell surface and fragmentation of the cell membrane. Consequently, it was noted that silver NPs efficiently inhibited bacterial proliferation. 

Similar to the previous example, another study assessed the antibacterial effectiveness of silver NPs, which were synthesized using silver nitrate and sodium borohydride along with starch as a stabilizing agent [337]. Characterized through diverse methods, the NPs showed strong antibacterial activity against both Gram-negative *E. coli* and Gram-positive *S. aureus*. The findings eventually confirmed silver NPs are effective across various conditions, promoting their potential in antibacterial applications

Like silver NPs, gold NPs have also become a major area of interest in NP studies. Although they are not efficient antibacterial agents on their own, their ease of functionalization makes them valuable materials in antibacterial research. As an example, a study investigated the antibacterial properties of gold NPs modified with phenylboronic acid derivatives. Researchers utilized thiol and amine groups, which have different binding affinities to gold, to anchor the phenylboronic acids. This modification enabled the NPs to selectively target and bind to specific molecules in bacterial cell walls such as lipopolysaccharide in Gram-negative and lipoteichoic acid in Gram-positive bacteria. Additionally, by altering the ratios of thiol to amine groups, they were able to customize the antibacterial activity of the gold NPs, making them adaptable for treating a range of bacterial infections, including those resistant to conventional antibiotics [338].

Additionally, gold NPs were synthesized using panchagavya (PG) as an eco-friendly reducing agent [339]. Synthesized PG–gold NPs were characterized and tested for their antibacterial efficacy against *E. coli*, *Bacillus subtilis* (*B. subtilis*), and *Klebsiella pneumoniae*. Findings indicated that PG–gold NPs exhibited significant antibacterial activity, particularly against Gram-negative bacteria. Furthermore, it was suggested that PG–gold NPs could be developed as alternative antibacterial agents against pathogens. 

Similarly to the referenced studies, metal ions such as maghemite and magnetite are also utilized in antibacterial research. For instance, IONPs are functionalized with citric acid to reduce agglomeration, representing a novel approach [340]. Particularly, NPs coated with a 0.3 M concentration of citric acid exhibited enhanced magnetic properties and demonstrated significant antibacterial activity, with a zone of inhibition measuring 36 mm.

Overall, considering the outcome of the referenced studies, a wide-ranging application of metal-based NPs in antibacterial research can be indicated.

Lastly, we discussed the diverse applications of semiconductor NPs in the previous sections. However, it should be mentioned that they also exhibit potential in the biomedical area as well. In particular, ZrO_2_’s low toxicity, compatibility with other materials, along with its antibacterial activity, further promote its employment in this area. 

In investigating the antibacterial activity of ZrO_2_ NPs, two bacteria strains, *S. aureus* and *E. coli*, were used and compared with ZrO_2_ complexes [341]. The antimicrobial screening showed that ZrO_2_ NPs exhibited activity against *E. coli*, but not against *S. aureus.* On the other hand, ZrO_2_ complexes not only showed activity against both bacterial strains but also displayed antifungal activity against five fungal strains. The researchers discussed that since chemical reactivity can be affected by the structure of the crystal, the difference in antibacterial activity could be due to the charge and arrangement of the components on the surface of the NP. Therefore, alterations on the surface of ZrO_2_ NPs can enhance antibacterial activity. A research study demonstrated an enhancement in the antimicrobial activity of ZrO_2_ NPs by biofunctionalization of the NP surface with glutamic acid [342]. The antimicrobial activity was tested against Rothia mucilaginosa, Rothia dentocariosa, Streptococcus mitis, and Streptococcus mutans. The results were compared with normal (pristine) ZrO_2_ NPs, and glutamic acid-modified ZrO_2_ NPs showed increased antimicrobial activity. The researchers suggested that the reason behind the enhancement is the change in the surface charge after the interaction of glutamic acid with the NP, potentially increasing the interaction between the NP and bacteria. It was also demonstrated that green synthesis of ZrO_2_ NPs can show antimicrobial activity [343].

#### 3.1.2. Inorganic Nanoparticles in Drug Delivery Systems

Inorganic NPs possess specific characteristics that make them favorable carriers in drug delivery systems. Their notably organized structure equips them with a high uptake capacity, incredible stability, and tolerably low toxicity and immunogenic responses [344].

AuNPs are favored due to their stability, biocompatibility, and the ability to be easily functionalized, which has induced their employment in various applications including drug delivery, cancer therapy, and biosensing [345]. In terms of drug delivery, AuNPs are also preferred, similar to other types of metal ions. For example, a study investigated the use of cyclic peptide-capped gold NPs (CP-AuNPs) as potential drug delivery systems. Cyclic peptides were synthesized acting as reducing and capping agents, efficiently forming CP-AuNPs. These NPs significantly enhanced the cellular uptake and retention of fluorescence-labeled antiviral drug lamivudine in cancer cells. Notably, lamivudine-loaded CP-AuNPs exhibited approximately 12 to 15 times higher cellular uptake than lamivudine alone, indicating the significance of utilizing gold NPs for enhancing drug delivery applications [346]. 

On the other hand, IONPs are highly valued for their superparamagnetic properties, large surface area, and chemical stability, making them ideal materials for drug delivery applications, as mentioned in the previous section. In a particular study, multifunctionalized IONPs were specifically designed for targeted drug delivery to CD44-positive cancer cells. The IONPs were coated with dimercaptosuccinic acid, to enhance stability, and functionalized with anti-CD44 antibodies along with chemotherapeutic gemcitabine derivatives. Results demonstrated that these IONPs can specifically target and deliver drugs to cancer cells overexpressing the CD44 receptor, which is a common marker for various cancer types [347].

In addition to metal ions, semiconductor NPs can also be effectively utilized in drug delivery applications. In a study, ZnO NPs were synthesized and then characterized to confirm their phase purity and structural properties. Next, they were encapsulated with chitosan to enhance drug loading efficiency and tested for their capability to effectively load and release ciprofloxacin, which is a commonly used antibiotic. Results demonstrated that the ZnO–chitosan complex showed significant initial release and high efficiency, highlighting the potential of ZnO NPs as effective carriers in drug delivery systems [348]. Hence, such an example involving ZnO NPs indicates the possible use of semiconductor NPs in advancing drug delivery technologies.

**Table 2:** Applications of inorganic NPs.

Type of the iNP	Application Area	Highlighted Results	References
Iron oxide	Drug delivery	- Successful delivery of anticancer drugs, sorafenib, doxorubicin, and ibuprofen, with enhanced activity - Modified Fe_3_O_4_ NPs possessed suitable magnetic field response, hydrophobicity, drug binding, and uptake for hypothermia therapy	[349,350,351,352]
Iron oxide	Bioimaging	- Improved efficiency and targeting for tumor diagnosis as a contrast agent for MRI (*in vitro*, *in vivo*) - Functionalized Fe_3_O_4_ NPs were utilized for NIR fluorescence imaging combined with MRI - Hybrid NP with gold and FeO_4_ used as a contrast agent in MR imaging	[353,354,355]
Iron oxide	Cancer treatment	- Modified Fe_3_O_4_ NPs synthesized into nanocomposite for efficient drug delivery and preventing breast cancer - Encapsulation of curcumin in chitosan-coated Fe_3_O_4_ NPs created a combined therapy, with increased drug effectiveness, against breast cancer - Polypyrrole–Fe_3_O_4_ NPs successfully tested against lung cancer by inhibiting metastasis and tumor growth with MRI capability (*in vivo*, *in vitro*)	[356,357,358]
Iron oxide	Antibacterial	- Synthesized iron oxide NPs from green sources possess significant antibacterial activity - Modified iron oxide NPs significantly prevented biofilm formation along with high stability and non-toxicity in cell culture	[359,360,361]
Iron oxide	Drug delivery	- Modified iron oxide NPs, carrying sorafenib, possessed significant magnetic properties and increased anticancer activity of the drug - Curcumin loaded and modified NP higher drug load capacity, cytotoxicity, and cellular uptake on cancer cells	[362,363]
Silver	Antibacterial activity	- Synthesis of Ag NPs from different sources possess significant bactericidal activity against both Gram-positive and Gram-negative bacteria - A combined structure of silver and copper exhibited extreme antibacterial activity when compared to their sole application	[364,365,366]
Silver	Drug delivery	- Silver NPs incorporated into Fe_3_O_4_ and SiO_2_ nanocomposite formed an efficient drug delivery system and tested for antibacterial and anticancer drug delivery - Silver NPs exhibited efficient release of phenindione and enhanced its anticoagulant activity	[367,368]
Silver	Anti-inflammatory activity	- Different source-derived silver NPs successfully modulate inflammation by suppressing pro-inflammatory cytokines (*in vitro*, *in vivo*)	[369,370]
Silver	Anticancer activity	- Green synthesized silver NPs show significant anticancer activity on liver and breast cancer cells (HepG2, MCF-7)	[371,372]
Silver	Food packaging	- A composite film, including silver NPs, extended the shelf life of carrots and exhibited significant antibacterial activity - Enhanced storage quality and antibacterial activity, and increased shelf life of tomatoes - Fabricated functional film coupled with silver NPs possessed significant antibacterial activity on foodborne pathogens, suitable water vapor permeability, and mechanical properties for food packaging	[373,374,375]
Gold	Antibacterial	- Gold–chitosan hybrid NPs were shown to be effective against antibiotic-resistant bacteria with desired stability and drug release of *Punicagranatum* L. extract - Gold NPs synthesized from different green sources exhibited great antibacterial activity	[376,377,378]
Gold	Drug delivery	- Gold NPs positively influence anticancer drugs by enhancing biodistribution, target-based routing, and release of the drug	[379,380]
SiO_2_	Industrial	- SiO_2_/nanodiamond hybrid NPs showed higher thermal stability, thermal conductivity and desired mechanical properties for tire tread production - SiO_2_/ZnO hybrid NPs were utilized for protection against acidic chloride-induced steel corrosion at 98.6% capacity - Usage of SiO_2_ NPs for the removal of zinc ions from wastewater greatly increased the removed ion percentage (66.58%)	[381,382,383]
ZrO_2_	Dental applications	- Green synthesized ZrO_2_ NPs showed significant antibacterial activity, along with an increase in osseointegration capacities and biointegration formation - ZrO_2_ NPs enhanced the mechanical properties of the 3D-printed resin, thus possessing potential as a provisional dental restoration - ZrO_2_ NPs encapsulated polyethyl methacrylate resin showed great antibacterial activity with a great potential to be used for provisional prosthesis	[384,385,386]
ZrO_2_	Antibacterial	- Dual-loaded antibiotics, ampicillin, and erythromycin possessed significant antibacterial activity, along with wound healing - Enhanced the antifungal and antibacterial activity of diamond-like carbon films by accumulating at the cell surface - Modified ZrO_2_ NPs with 3D-printed resin managed to show significant antibacterial activity and antibiofilm formation without any side effects	[387,388,389]
ZnO	Food packaging	- Chitosan-based developed ZnO NPs, capsulated with gallic acid films, significantly increased antioxidant, antibacterial, and mechanical properties of chitosan film for food packaging - ZnO NP-based food packaging films were developed; mechanical and water vapor barrier properties were enhanced along with high antimicrobial activity against food-borne pathogens - A hybrid NP with chitosan loaded with clove essential oil was formed and acted as a strong barrier against UV rays, oxygen, and water vapors, along with showing antimicrobial and antioxidant activity for extending the shelf life of chicken meat	[390,391,392]
ZnO	Antibacterial	- Synthesis of ZnO NPs from different sources, such as leaf extracts or other microorganisms, exhibits significant antibacterial activity	[393,394]
ZnO	Cosmetics	- Sodium and aluminum-doped ZnO NPs were synthesized as a UV filter sunscreen cream significantly prevented photocatalytic activity and showed great antioxidant capacity	[395]
ZnO	Cancer	- Significant cytotoxicity was observed with increasing intracellular ROS levels on MCF-7 breast cancer cells - Anticancer activity of the ZnO NPs on HeLa cells was demonstrated with apoptosis induction mechanism for cervical cancer - Similar apoptosis induction against different types of cancer cells was also conducted (such as colorectal and liver)	[396,397,398,399]

#### 3.1.3. Inorganic Nanoparticles in Food Packaging and Preservation

In food packaging, the biodegradability, molecular structure, and toxicity of the main nanocompound in the packaging are crucial factors. Given that the product inside the package will constantly interact with it, some part of the package will inevitably migrate into the product [400]. This means that the used material in the package will be ingested when the product is consumed. In these terms, the base material for the package should be biodegradable and non-toxic to avoid any unwanted results in the long term. The biodegradability and non-toxicity of polymers, especially chitosan, are primary traits in their application on food packaging, as covered in the organic NP application section. 

Food packaging and preservation focus on similar challenges; the packaging material should be non-toxic and highly biodegradable, as mentioned, for the sake of both food consumption and the environment. Meanwhile, food preservation also focuses on the condition of the food for consumption, but it mainly aims to prevent spoilage of the food at the highest capacity. Nanostructure-based approaches are highly favored in food packaging and preservation, given their ability to deal with microbial spoilage, along with other addressed challenges [401]. In this case, materials that can show high antibacterial activity are usually preferred in NP-based food preservation studies.

We have discussed the usage of chitosan in food packaging and preservation in the following sections, addressing both aspects. Chitosan’s preferability as a primary material for enhancing the application of inorganic NPs is apparent in numerous studies, and some of them have been mentioned in Table 2. Hereby, chitosan is utilized with inorganic NPs in food packaging studies. For instance, similar research using silver NPs was conducted with chitosan to create an antibacterial film for food packaging [402]. The film was created using chitosan as a stabilizer and polyvinyl alcohol for biofilm formation with silver NPs. Characterization of the biofilm revealed that the addition of silver NPs enhanced water resistance and increased tensile strength through hydrogen bond formation, although higher concentrations showed a converse effect. In addition, antibacterial tests against *E. coli* and *S. aureus* yielded significant results, successfully preserving strawberry samples for day 9 at certain concentrations. Another study utilized silver NPs with microcrystalline cellulose, as well as starch and whey protein [403]. The researchers synthesized silver NPs using leaf extract from Azadirachta indica, demonstrating a form of green synthesis. Antibacterial tests were conducted using two Gram-positive (*S. aureus* and *Bacillus cereus*) and three Gram-negative (*E. coli*, *Pseudomonas aeruginosa*, and *Proteus vulgaris*) bacteria. The silver NPs showed significant activity against all bacteria except Proteus vulgaris. Eggplant was chosen for the preservation test, and biofilm coating preserved the vegetable for 15 days. The significant antibacterial characteristics of silver NPs are a primary factor in their preference for this application, as discussed above.

Additionally, other types of metal ions are commonly used for food preservation. For instance, a packaging film was created using gold NPs combined with polyvinyl alcohol to increase the shelf life of banana fruit [404]. Graphene NPs were also synthesized, tested, and compared with gold NPs. The analysis of mechanical properties showed that both graphene and gold NPs increased tensile strength and Young’s modulus but decreased elongation at break. In addition, gold NP-based film showed the lowest water transmission rate and the highest antibacterial activity against *E. coli*. In the end, the film with the gold NP showed the best preservation of banana samples on day 5. A similar food packaging study was conducted using green biosynthesized copper NPs with cellulose acetate and polycaprolactone [405]. Likewise, usage of copper NPs decreased the water transmission rate, increased tensile strength, and showed significant antimicrobial activity against several bacterial and fungal strains: *E. coli*, *S. aureus*, *Candida albicans*, *Aspergillus niger*, *Penicillium expansum*, and *Fusarium oxysporum*.

Apart from the metal ion-based NPs, other subclasses of inorganic NPs are also applied in food application and preservation. Semiconductor NPs are also known to be effective in food packaging. For instance, the current literature includes many studies of ZnO NPs in food packaging studies to enhance previously referred properties such as water transmission, antimicrobial, mechanical, and so on [406]. As an example, an antimicrobial biofilm was synthesized for food packaging, combining ZnO and silver NPs [407]. During the examinations, ZnO NP managed to absorb UV light and scatter visible light. The values of elongation at break and tensile strength were observed, depending on the percentage of silver and ZnO in the NPs. The single usage of ZnO and silver NP possessed similar values, but when the proportion of silver was higher in the combination, both values increased significantly. As most of the food packaging studies tested, the antibacterial activity experiments against *E. coli* and *S. aureus* were conducted. The antibacterial activity against *S. aureus* was higher than the *E. coli* due to the cell wall difference between the bacteria, as was mentioned by the researchers. Although silver NPs showed higher activity, the addition of ZnO to the film increased the activity significantly.

To avoid repetition, we will include one final example of NP application in this section to highlight the role of ceramic NPs. A recent study used TiO_2_ NPs with low-density polyethylene to test their effectiveness in food packaging [408]. An increase in the concentration of TiO_2_ NPs in the nanocomposite greatly reduced the water vapor transmission rate but subsequently increased the oxygen transmission rate. The authors commented on these results by suggesting that the use of TiO_2_ may be preferred for packaging certain types of foods. The mechanical tests showed an increase in tensile strength, which is not unusual, but a decrease in elongation at the break value. Finally, *P. aeruginosa* and *S. aureus* were used for an antibacterial test, which was successful, and the antibacterial activity slightly increased proportionally to the concentration of TiO_2_ NPs.

Most subclasses of inorganic NPs, particularly metal NPs, are used in food packaging and preservation. We provided several examples of NP applications in food packaging, including metal, semiconductor, and ceramic NPs. Additional examples are listed in Table 2. When the discussed studies are thought of, the antibacterial activity, mechanical strength, and transmission of water and oxygen are the primary factors that have been observed during experiments. In this case, the abundance of studies with inorganic NPs in the application of food preservation is quite expected, especially when the common characteristics of NPs are considered. Since most of the inorganic NPs are significant antibacterial agents, and they all share NP traits, it is possible to say that they are quite preferable in this area. They are not the only type of NP that is preferred in this area, since some of the subclasses of organic NPs are also known for antibacterial activity and possess high biodegradability, as we discuss in later sections.

#### 3.1.4. Inorganic Nanoparticles in Cosmetics 

TiO_2_ and ZnO NPs are commonly used in cosmetics, particularly as sunscreens, due to their ability to filter UV light [409]. They can absorb and/or reflect UV photons, depending on their particle size [410]. This is why they have been widely used in sunscreen products for a long time. One common disadvantage that is discussed and studied is the photoactive nature of TiO_2_ NPs. TiO_2_ can initiate synthesis of ROS when it is photoactivated, which is considered as a general problem for its safety [411]. The general approach to prevent ROS synthesis is to combine TiO_2_ NPs with other molecules that can scavenge these reactive molecules, as explained in one of the studies discussed in the carbon-based NP cosmetics section. However, with the application of ZnO NPs as a sunscreen ingredient, there is a possibility that it can cause slight toxicity under high concentrations. Studies have shown that this can be prevented by using TiO_2_ NPs simultaneously [412].

Since both of these molecules have proven their efficiency and are often studied to compare new methods in their characterization and reduce their potential toxicity, a few examples will be given to emphasize and discuss their protection against UV light. To give an example, the protection of TiO_2_ NPs against UV irradiation was investigated to determine the effect of particle size on activation [413]. As the activity of TiO_2_ NPs was affected by size, three different sizes of NPs were used in the experiment (the size of the particles was decreased to optimal nanometers during the research). The smallest particle showed higher dispersion and UV protection and pointed out its suitability for sunscreens. Another study used ball-milling to enhance the UV protection activity of both TiO_2_ and ZnO NPs when mixed [414]. Three types of samples were used and compared: without being milled, one-time milled, and two-time milled. Each sample was used to formulate a sunscreen cream, and a two-time milled mix showed the highest UV absorption rate. The produced free radicals were significantly lower in two-time milled samples, compared to the rest. In addition, it was shown that visible light absorption was lower in the milled groups (which is considered an advantage rather than a disadvantage due to the increased transparency). This was, however, not the case for the UV absorption, which was statistically not significant when compared among each other. To give one last example, a TiO_2_-ZnO composite was synthesized for enhanced UV absorption [415]. Referring to the first study discussed in this section, TiO_2_’s particle size was also a factor that affected the UV absorption; meanwhile, the particle size of the ZnO did not alter the absorption capacity. Most importantly, the new composite showed higher overall UV absorption, contributing to research that aimed at improving the application of both of these NPs.

TiO_2_ and ZnO NPs have remarkable potential in the cosmetics industry thanks to their significant properties. The recent literature researches how to improve the efficacy and safety of these two NPs. Much important research currently exists that compares the methods, size of the particle, novel characterization methods, and so on. Nevertheless, we briefly discuss their key properties to highlight the role of inorganic NPs in cosmetics.

### 3.2. Carbon-Based NP Applications

Carbon-based NPs possess unique traits that allow for them to be utilized in distinct areas as an NP and supportive material.

#### 3.2.1. Carbon-Based NPs in Tissue Engineering

NPs are prominent nanostructures included in tissue engineering studies and applications. Scaffolds are 3D materials that provide mechanical support and facilitate cellular interactions for proliferation and cell attachment in tissue regeneration [416]. NPs are primarily highlighted in scaffold applications to enhance mechanical support and the bioactivity, release, and solubility of bioactive molecules [417]. Carbon-based NPs are considered in this area to enhance the stability and mechanical properties. For instance, GO NPs are used for mechanical support and to increase proliferation rates. GO itself is widely involved in many nanocomposites, often with other NPs for similar purposes. Conversely, CQDs are used for antibacterial support and tissue regeneration. For simplicity, we discuss CQD and GO NPs in this section to highlight the involvement of carbon-based NPs in tissue engineering, with additional studies provided in Table 3.

CQD-co-incorporated GelMA hydrogel scaffolds were investigated for their use in treating bone defects infected by multidrug-resistant bacteria, with their surface charge being regulated. Positively charged CQDs exhibited noteworthy antibacterial activity against *E. coli*, *S. aureus*, and MRSA, a multidrug-resistant bacteria, and also prevented biofilm formation. On the other hand, negatively charged CQDs significantly promoted bone regeneration, nearly achieving complete repair of bone defects at the end of 60 days [418]. To give one additional example, different nanocomposite scaffolds were created with CQDs, poly glycerol sebacate, and polycaprolactone for cardiac tissue engineering [419]. Various aspects of the synthesized nanocomposite were investigated, including mechanical properties, cell viability, conductivity, and compatibility. The addition of CQDs affected the application of the nanocomposite by increasing Young’s modulus, electric conductivity, fiber arrangements, and cell proliferation. Additionally, a high percentage of CQD in the nanocomposite had an adverse effect on cell viability by slightly decreasing it (0.5 wt% chosen as the optimum ratio). 

As mentioned, GO NPs are widely included in many nanocomposites for tissue engineering, similar to CQDs. As an example, a study synthesized GO NPs with a chitosan scaffold to build up a nanocomposite for cartilage tissue engineering [420]. Regarding the significant mechanical properties of GO, it was aimed to enhance the scaffold mechanically. As a result, the addition of GO NPs in certain concentrations (up to %2 W/V) increased mechanical properties in decent amounts. Subsequently, the proliferation of chondrocytes for cartilage engineering was evaluated. Based on the NP concentration, the scaffold including GO showed the highest rate of proliferation, with GO acting as an additional proliferation promoter. Similarly, silk fibroin/soy protein scaffolds were utilized with β-tricalcium phosphate and GO NPs to enhance mechanical properties and osteoinductivity [421]. The initial observation was a significant increase in compressive strengths due to the addition of GO NP and β-tricalcium phosphate to scaffolds. For determining the efficiency of the scaffolds in bone engineering, the biomineralization capability of each group was tested. The accumulation of minerals on the surface of each type of scaffold was observed for two weeks. Visuals acquired by electron microscopy showed that GO NPs increased aggregation of the minerals and mineral deposition of the scaffold. Later, proliferation comparisons of the scaffolds were observed in the cell viability test. Not only was the proliferation rate highest in the GO NP-added scaffolds (especially on days 5 and 7), but also it showed the best morphological results during the examination. Finally, to observe the enhancement in proliferation from another aspect, osteogenesis-related gene expressions were analyzed. On both days (7 and 14), the scaffold that included GO NPs showed the highest mRNA expression rates.

#### 3.2.2. Carbon-Based NPs in Antitumor and Cancer Research

Carbon-based NPs have drawn considerable attention in antitumor and cancer research. Along with the direct interaction with cancer cells, carbon-based NPs are commonly modified to shape their application in targeting and delivery systems. This capability of modification of carbon-based NPs enhances tumor targeting and antitumor activity, making them valuable in cancer research [422]. Fullerene’s wide range of applications in delivery systems, light-sensitive ROS generation, and anticancer activity are the major factors behind its application in antitumor and cancer research.

For instance, a study used fullerene C_60_ NPs to test out antitumor activity on breast cancer in both *in vitro* (MCF-7 cells) and *in vivo* (Wistar albino rats) [423]. During the *in vivo* experiment, specific parameters were identified and compared to the effect of C_60_ after the induction of breast cancer. The results showed that the addition of C_60_ after induction managed to decrease the oxidative stress and toxin levels in breast tissue, and increased catalase activity, glutathione levels, and protein density. Afterward, the histological images pointed out positive results on behalf of C_60_, as the tumor diameters were lower in the fullerene group. On the other hand, *in vitro* experiments showed a dose-dependent activity (demonstrating activity at 25 mg/mL and higher) of C_60_ in a cell viability test. 

In addition to the direct application of C_60_ in cancer research, it is also applied for cancer cell imaging and tumor targeting. In an experiment, highly fluorescent fullerene C_60_ NPs were synthesized, modified with folic acid, and used for cancer cell imaging [424]. Folic acid’s specific affinity against folate receptors, which is found in high levels in cancer cells, was utilized with fullerene’s luminescent characteristics. To determine any potential cytotoxicity, cell viability tests were conducted using COS-7 cells, which showed safety for administration up to 200 μg/mL. Later on, COS-7, U87, and HeLa cells were used for testing the selective imagining property of C_60_ NPs. U87 and HeLa cells showed blue fluorescence; meanwhile, COS-7 did not show any, confirming its selectivity. A similar study used C_60_ NPs for tumor targeting to enhance antitumor activity [425]. During the experiments, a delivery system known as an “off-on” state was developed, and doxorubicin was used as the anticancer drug. The “off” state represents the encapsulated drug’s protection until it reaches the site of action, where it transitions to the “on” state for instant drug release. Doxorubicin was covalently attached to the C_60_ NP via ROS-sensitive linkage, representing the “off” state. To activate drug release (representing the “on” state), the light sensitivity of C_60_ was activated (by laser irradiation), inducing ROS generation to break the ROS-sensitive linkage and release the drug immediately. This experimental model was applied in both *in vivo* and *in vitro* experiments. For the *in vitro* experiment, 4T1 cells were used, confirming the tumor-targeting capability of C_60_ NPs and showing a decrease in cell viability after entering the “on” state. Finally, an *in vivo* mice study demonstrated significant antitumor activity and reduced cytotoxicity compared to administering the drug alone, thanks to the tumor-targeting enhancement.

Another study also investigated C_60_ in a cancer study but from a different perspective. C_60_ NPs were tested on rats against induced hepatotoxicity by a chemotherapeutic agent, cyclophosphamide [426]. To evaluate the effect of C_60_ against hepatotoxicity, five groups were created and orally administered C_60_ (alone or with zinc chloride). First, the levels of liver enzymes and liver disease markers in blood plasma were determined for all groups. C_60_ prevented the increase in marker levels and significantly reduced the increase in liver enzymes to levels near those of the control group (further enhancement was observed with the addition of zinc chloride). Further, C_60_ and zinc chloride increased the low levels of albumin and protein levels to nearly control levels. The significant antioxidant activity of C_60_, well known in the literature, was also observed during the experiments, as indicated by decreased oxidative stress markers and increased glutathione levels. These results further indicate the potential of C_60_ NPs in cancer research, particularly as agents to relieve the side effects of cancer treatments.

Similarly, CQDs also play a significant role in cancer research. Since they possess significant photostability, fluorescence, and compatibility, CQDs are applied in various applications related to cancer treatment and diagnosis, such as photoinduced therapy, tumor inhibition, drug delivery, and fluorescence imaging [427,428].

For instance, a study investigated the development of a novel drug delivery system involving the integration of the anticancer drug 5-fluorouracil (5-FU) with CQDs [429]. For confirming the successful characterization of 5-FU-CQD nanoconjugate, various tests such as drug release kinetics and cytotoxicity assays were applied to evaluate its potential in drug delivery for improving the efficiency and security of cancer treatment. Results indicated the 5-FU-CQDs exhibited enhanced drug delivery capabilities, increased cytotoxicity against cancer cells, and improved cellular uptake compared to the free drug when tested on normal human lung fibroblast (GM07492A) and human breast cancer (MCF-7) cell lines. Overall, these findings suggested a promising approach to advanced cancer therapy. In addition, a study evaluated the cancer cell imaging property of CQDs modified with folic acids [430]. HeLa and MCF-7 cells were used to test and perform fluorescence imaging. Before that, the cell viability of CQD was checked with MCF-7 cells and showed no morphological damage during the administration. The imaging results showed that modified CQDs showed significant photoluminescence; meanwhile, unmodified CQDs showed a total opposite. Although CQD can target cancer cells with several approaches, functionalization of this NP indeed yields more promising results [431].

**Table 3:** Applications of carbon-based NPs.

Type of the Carbon-Based NP	Application Area	Highlighted Results	References
Fullerene	Antioxidant	- Enhancement of antioxidant capacity by increased catalase activity (*in vivo*) - Protection against oxidative cell injury	[432,433]
Fullerene	Diabetes	- Antioxidant activity and tissue protection in hyperglycemia-induced lung damage (*in vivo*)	[434]
Fullerene	Anti-inflammatory	- Potential anti-inflammatory activity against heart tissue damage by inflammation *(in vivo*) - Reduction in inflammatory responses in eye tissue by inducing HO-1 protein expression (*in vivo*)	[435,436]
Fullerene	Drug delivery	- A fullerene C_60_-included conjugate was functionalized and successfully conducted to efficiently target tumor cells for delivery applications	[437]
Fullerene	Cancer	- Fullerene C_60_ NPs were tested for photodynamic therapy against a human lung cancer line and possessed great potential for future administrations	[438]
Graphene	Antibacterial	- Enhancement in antibacterial activity in copper and silver NPs - Enhancement in bacterial detection and antibacterial activity with gold NPs	[439,440]
Graphene	Drug delivery	- Increased targeting and drug release in anticancer drug delivery systems - Efficient anticancer drug delivery with enhanced accumulation	[441,442,443]
Graphene	Anticancer	- Hybridization with gold NP for enhancing cancer imagining and anticancer activity (*in vivo* and *in vitro*) - Decoration on CuO NPs for enhancing stability and anticancer activity	[444,445]
Graphene	Industrial	- Enhancing mechanical properties of materials - Enhancing gas-sensing traits of TiO_2_ NPs - Material for enhanced thermal energy storage and water production	[446,447,448,449,450]
Graphene	Tissue engineering	- Graphene-included nanocomposite exhibited significant antibacterial activity and increased proliferation of mesenchymal stem cells for bone tissue engineering - Chitosan and graphene-included scaffolds increased viability of stem cells, enhanced biodegradation, and improved cell–scaffold adherence for neural tissue engineering - Reduced GO and alginate-included nanohybrid nanogels improved thermal stability, increased myoblast adhesion and myogenic differentiation, and enhanced physical and chemical properties of the structure for skeletal muscle tissue engineering	[451,452,453]
Carbon black	Electrochemical sensor design	- A biosensor based on the change in algae oxygen evolution from herbicide exposure was developed - Modification of electrochemical sensor by Super-P CBNP enhanced the detection of carbendazim by improved efficiency of charge transfer	[454,455]
Carbon black	Reinforcement of cement-based materials	- Up to 3% addition of CBNPs increased compressive strength, electrical resistivity, and static modulus of cement mortars (addition of CBNPs higher than the 3% showed converse effect) - A generated cement–CBNP enhanced energy conversion performance of a cement-based nanogenerator	[456,457]
Carbon black	Energy storage	- Functionalized form enhanced cell performance, ion selectivity, and stability in vanadium redox battery. - Synthesized hollow carbon NPs with carbon black significantly improved electrolyte storage, capacitive, and rate performance.	[458,459]
Carbon quantum dot	Bioimaging	- Exhibited characteristics, cell penetration and fluorescence emission, that are suitable for bioimaging applications - Significant fluorescence image, decent biocompatibility, no significant toxicity was observed (*in vivo*) - Enhanced light stability and photobleaching resistance in bacterial bioimaging application with specific staining	[460,461,462]
Carbon quantum dot	Biosensor design	- CQD–polyaniline biocomposite biosensor successfully managed to detect low levels of dopamine - A more specific, sensitive, and ranged fluorescent biosensor for acrylamide detection was developed - Real-time and sensitive fiber optic biosensor based on CQD successfully developed for nitric oxide detection - CQD–chitosan composite used for sensitive, selective, and cost-efficient detection of insulin.	[463,464,465,466]
Carbon quantum dot	Antibacterial	- CQD exhibits significant antibacterial activity, increases the recovery of infected wounds, and decreases infection death (*in vivo*) - CQDs avoid antimicrobial resistance, generate ROS, and possess intracellular antibacterial activity - Combined application of CQD with different types of NP shows enhanced antibacterial activity	[467,468,469]
Carbon quantum dot	Drug delivery	- CQD-included hybrid NP successfully delivered 5-fluorouracil for breast cancer treatment - Combination of CQDs and PLGAs successfully carried two types of antibiotics, azithromycin, and tobramycin, with significant antibiofilm activity - Cytarabine-encapsulated CQDs, in combination with chitosan gels, possessed pH-sensitive drug release with high efficiency	[470,471,472]
Carbon quantum dot	Tissue engineering	- The addition of CQD NPs to the bioactive scaffold increased the expression of cardiac-marker genes and the value of Young’s modulus - The addition of CQD NP into the PLGA scaffold significantly increased osteogenesis, bone mineralization, and proliferation for bone tissue engineering	[473,474]
Carbon quantum dot	Wound healing	- Nitrogen-doped CQD-included nanocomposite, along with several biomolecules, used for wound healing and skin tissue regeneration by exhibiting high antibacterial activity and great Young’s modulus and tensile strength values - CQDs modified with various components promoted *in vivo* wound healing	[475,476,477]

#### 3.2.3. Carbon-Based NPs’ Antioxidant Activity and Cosmetic Applications

The radical scavenging activity of fullerene is well known and distributed across multiple fields, including cancer research and dermatology. To emphasize, the primary reason that fullerene is widely involved in cosmetics and dermatological research is its characterization as a “radical sponge” [478]. The antioxidant activity of C_60_ is not limited to the general radical scavenging studies. Its conditional ROS-generating characteristics and possible mitochondria-targeted antioxidant activity are an interesting subclass within this research area. 

As an example, an experiment was conducted for the antioxidant activity of C_60_ NPs combined with polydopamine and glutathione via Michael’s addition reaction [479]. An *in vitro* radical model was used to test the radical scavenging activity of the combined C_60_ NPs. Dose-dependently, combined C_60_ NPs scavenge up to 86.2% of the hydroxyl radicals at 200 μg/mL. Considering the light-sensitive ROS generation trait of C_60_, a cell viability test was conducted in the presence and absence of light. In the dark, cell viability values were slightly higher, indicating the potential proliferative activity of this NP. Conversely, under white light irritation, nearly all cell viability values matched the control group, indicating the intensity of the ROS scavenging ability compared to ROS generation with negligible cytotoxicity. The experiment progressed by testing the oxidative stress levels in cell cultures: HEK-a, HUVEC, HM, and L-02 cells. At 20 and 30 μg/mL, the cell viability of all types of cells was higher, with a mean value of 80% viability. Finally, the observed cellular uptake results showed that C_60_ NPs are localized in the mitochondria. Fullerene’s possible mitochondrial localization and antioxidant activity are discussed in another study [480]. This research investigated the two-edged activity of C_60_ NPs using an *E. coli* model. The antioxidant and prooxidant activities of C_60_ were investigated with multiple tests. C_60_ NPs concentration-dependently exhibited intense protective activity but shifted towards prooxidant activity after nearly 50 min. At certain concentrations, especially 10^−3^ and 10^−4^ g/L, the highest protective activity was observed, along with the least prooxidant activity. The *E. coli* strain used is known for exhibiting its antioxidant system and enzymes, similar to mitochondria. Following this correlation, this model can meet the specifically directed antioxidant ability of C_60_ toward mitochondria.

As discussed, thanks to the significant antioxidant activity of C_60_ NPs, they have a wide range of applications in the current research. Related to its antioxidant activity, C_60_’s protective effect against UV-induced damage is also a major trait in dermatological applications. For instance, the UV-protection and antioxidant activity of C_60_, combined with nanodiamonds (NDs) was evaluated for sunscreen formulation [481]. As discussed in the previous section, ZnO and TiO_2_ NPs are used in UV protection and cosmetics. This study investigates the addition of C_60_ and NDs to TiO_2_ NPs for sunscreen formulation, providing supportive protection against ROS generation by TiO_2_ during photoactivation. In addition, the scavenging potential of vitamin C, C_60_, and NDs was determined and observed in the presence of TiO_2_ (under dark lighting conditions). After vitamin C (since it has the lower molar mass value), C_60_ showed the highest potential (per unit mass), followed by the NDs. The presence of TiO_2_ in the dark did not disrupt the scavenging potential of any compounds. During the irradiation, vitamin C showed a decrease in its scavenging potential, possibly due to activated ROS synthesis by TiO_2_ NPs. On the other hand, both C_60_ and the NDs showed increased scavenging activity (higher than in the non-irradiation test), even after the photoactivation of TiO_2_ NPs. However, C_60_ did not decrease intracellular oxidative stress levels, as it can generate ROS under irritation as well. In addition, both C_60_ and NDs decreased the transmission of UV light and showed anti-UV traits. The absorption of UV photons by TiO_2_ was also demonstrated.

#### 3.2.4. Carbon-Based NPs’ Anti-Inflammatory Activity

Fullerenes, particularly C_60_, have gained attention in anti-inflammatory research due to their significant radical scavenging capability, resulting from their conjugated double bonds and high electron affinity [482]. These carbon-based NPs exhibit antioxidant properties, allowing for them to neutralize ROS and free radicals that contribute to inflammation and cellular damage, thereby reducing oxidative stress and associated inflammatory responses [483]. Regarding these characteristics, multiple studies in the current literature have explored the potential of fullerenes in anti-inflammatory studies. 

To exemplify, a study investigated the therapeutic potential of n C_60_, the aqueous suspension form of C_60_, in treating atopic dermatitis in mouse models [484]. Researchers observed that n C_60_ significantly reduced IgE production and Th2 cytokine levels, particularly IL-4 and IL-5, while inducing a Th1 immune response with increased concentrations of IL-12 and IFN-γ. Additionally, epicutaneous administration was more effective than subcutaneous administration, evidenced by a considerable increase in filaggrin expression and a notable reduction in eosinophil and leukocyte infiltration. Overall, n C_60_ holds potential as a promising therapeutic agent for the treatment of atopic dermatitis by modulating immune responses and enhancing skin barrier functions. 

Similarly, in another study, researchers used C_60_ fullerene suspension (C_60_FS) to treat ulcerative colitis (UC), a chronic inflammatory bowel disease characterized by ulcers in the colon [485]. *In vivo* experiments on rats showed that C_60_FS repaired barrier dysfunction in the colon, reduced inflammation, promoted ulcer healing, and enhanced colon health compared to the group treated with mesalazine enema (ME), a commonly used anti-inflammatory drug for UC. Moreover, C_60_FS decreased the number of mast cells and basophils, thereby suggesting a broader anti-inflammatory effect. In summary, these findings collectively highlight that C_60_Fs can be an effective therapeutic agent for treating UC, potentially offering better outcomes compared to conventional medications like ME.

Besides fullerene, CQDs have also been used as anti-inflammatory agents in various studies. For example, recent research demonstrated the effectiveness of CQDs, derived from *Carthamus tinctorius* L. and *Angelica sinensis*, in reducing inflammation associated with rheumatoid arthritis (RA) [486]. *In vivo* experiments on rat models revealed that CQDs exhibited remarkable anti-inflammatory properties, effectively reducing inflammation. Additionally, CQDs derived from *C. tinctorius* and *A. sinensis* significantly downregulated pro-inflammatory cytokines such as IL-1, IL-6, and TNF-α, as well as vascular endothelial growth factor (VEGF), which are known to play crucial roles in the pathogenesis of RA.

#### 3.2.5. Carbon-Based NPs in Diabetes

Due to their proper molecular structure and superior antioxidant activity, fullerene NPs are studied with *in vivo* experiments on diabetes and diabetes-related abnormalities [487]. To illustrate the direct activity of fullerene NPs against diabetes, an *in vivo* study demonstrated the potential of fullerene C_60_ NPs in diet-induced obesity in rats [488]. For 70 days, rats were fed a normal and high-fat diet separately, with C_60_ NPs administered after day 28. As expected, the high-fat-fed rat group showed significantly higher weight, insulin, glucose, and pro-inflammatory cytokine levels compared to the control, low-fat diet group. The administration of C_60_ NPs decreased the mentioned metabolic parameters to control levels, slightly reduced body mass index (from 2.7 to 2.3 times), and normalized anti-inflammatory cytokine levels. In addition, increased oxidative stress caused by the fat diet was also reduced by C_60_, followed by increased antioxidant enzyme activity.

C_60_ NPs, along with their diabetes-regulating properties, are also used to encapsulate certain molecules for diabetes treatment. As an example, a study demonstrated the combination of curcumin and C_60_ fullerene NPs against kidney damage in *in vivo* diabetes rats [106]. The kidney tissue of the diabetic rats, divided into nine groups, was examined. The administration of curcumin, C_60_ NPs, and a combination of these two was compared to observe the kidney damage. The results indicated that the combined treatment increased the protection of kidney tissue by reducing oxidative stress, fatty acid, and cholesterol levels. In recent years, the same researcher tested the potential protective activity of C_60_ fullerene NPs against pancreatic damage in an *in vivo* rat study, which showed promising results [489]. To give one last example, fullerene NPs, attached to porphyrin and encapsulated with magnesium-25, were tested on diabetic neuropathy [490]. In an *in vivo* experiment, the changes in the numbers of small and large neurons were investigated and compared between control and NP-treated groups. In the diabetic neuropathy group, the number of large neurons was halved, and the number of small neurons doubled compared to the control group. The NP group significantly increased the number of large neurons and decreased the number of small neuron numbers back to control levels when compared to diabetic neuropathy-induced rats. Additionally, similar to other studies, increased antioxidant capacity and a decrease in lipid peroxidation were also observed in the NP-treated group. At last, each group’s motor function was tested, and NP groups showed very near results compared to control groups.

#### 3.2.6. Carbon-Based NPs in Drug Delivery Systems

Due to their significant optical absorption, stability, and reactive surface area, resulting from the characteristics of the carbon atom, carbon-based NPs have an important place in drug delivery studies [491]. GO-based nanomaterials (including NPs) possess significant characteristics, especially their unique electrochemical nature; they have a remarkable potential in drug loading and delivery [492]. Nevertheless, they have been primarily investigated and discussed for their potential as another type of NP suggested in drug delivery systems.

As an example, in an experiment, GO NPs were functionalized with PEG to deliver an antibacterial *Nigella sativa* extract [493]. To test the antibacterial activity of the system, two types of bacteria, *S. aureus* and *E. coli*, were used along with two additional groups: GO NPs and GO NPs-PEG. In both types of bacteria, each group showed significant antibacterial activity, but the group that included Nigella sativa showed the highest activity, as expected. In addition, all groups promoted the generation of ROS in significant amounts, indicating the GO NPs’ antibacterial activity together with its suitability for drug delivery systems. A related study used PEGylated GO NPs to enhance the anticancer activity of doxorubicin and cisplatin [494]. First, the drug loading and drug release potential of the NPs were tested. The characterization of drug loading showed that GO NPs loaded cisplatin in superior amounts, by exceeding levels of other types of NPs that are used in similar approaches. Thereafter, GO NPs showed pH-sensitive drug release, which is desirable in common anticancer deliveries for releasing the drug at tumor sites. Secondly, both drug carrier and non-carrier forms of PEGylated NPs were investigated with cell viability and cytotoxicity tests with CAL-27 and MCF-7 cells. GO NPs showed cell viability higher than 90%, confirming their safety in the delivery system. As expected, drug-loaded GO NPs showed significant cytotoxicity and apoptosis rates. Also, the simultaneous delivery of doxorubicin and cisplatin showed the highest activity compared to single-drug delivery. At last, an *in vivo* experiment was designed to determine the antitumor efficacy. The dual drug delivery of doxorubicin and cisplatin was applied with and without the GO NPs. It was observed that even though the dose concentration was 2–3-fold higher in the non-capsulated drugs, the encapsulated GO NP group showed higher accumulation in tumor cells.

Similarly, CQDs are also included in some drug delivery systems, particularly in cancer research. Since they possess various functional groups and can be functionalized with diverse ligands, CQDs have become one of the most discussed types of NPs in cancer drug delivery [431]. For instance, researchers aimed to develop targeted drug delivery systems, particularly for breast cancer therapy, by employing transferrin-conjugated CQDs (TF-CQDs) loaded with doxorubicin [495]. *In vitro* experiments showed that Dox-loaded TF-CQDs reduced cell viability in the MCF-7 cell line and enhanced cellular uptake more effectively compared to doxorubicin (Dox) or TF-CQDs alone. Moreover, it was stated that the pH of the environment played a significant role in the release of Dox from TF-CQDs, with acidic conditions enabling a more effective release. The delivery of Dox with CQDs was evaluated similarly to cancer stem cells [496]. HeLa and breast cancer stem cells were used in the *in vitro* experiment. For both types of cells, it was observed that CQDs managed to penetrate the cell membrane and accumulated in the cytoplasm and nuclei of the cells. Later, the same type of visualization was performed *in vivo* on mice. After 12 h, there was a significant accumulation of CQDs in the tumor sites with strong fluorescence. Furthermore, the emission intensity of the CQDs did not change after day 10. Compared to free Dox administration, CQDs showed significant potential and enhancement in drug delivery.

#### 3.2.7. Carbon-Based NPs in Antibacterial Research

Carbon-based NPs have been studied for their antibacterial properties. Among the carbon-based nanomaterials, graphene and carbon dots exhibit several antibacterial mechanisms, such as directly damaging the cell wall of the bacteria, generating significant amounts of ROS, and possessing inhibitory effects on bacteria metabolism [497]. In addition to these chemical and physical antibacterial mechanisms, they are also combined with other NPs, and functionalized with distinct molecules due to their physical properties [498].

As an example, researchers demonstrated phosphorus-doped CQDs (P-CQDs) possess bactericidal effects on both *E. coli* and *S. aureus*, with minimum inhibitory concentrations measured at 1.23 mg/mL and 1.44 mg/mL, respectively. The synthesized P-CQDs underwent characterization steps to confirm their composition and properties. Following the experiments, it was observed that morphologies of *E. coli* cells were damaged and also, *S. aureus* became irregular when treated with P-CDQs, indicating their potential as effective antibacterial agents [499].

Another study, focused on the potential of CQDs derived from curcumin as antibacterial agents [500]. It was shown that CQDs functionalized with quaternary ammonium groups (Q-CQDs) exhibited strong binding to bacterial membranes, facilitating membrane disruption, ROS generation, and subsequent cell death. In addition, *in vitro* and *in vivo* assays indicated that Q-CQDs successfully eliminated both *S. aureus* and *E. coli* and facilitated wound healing while exhibiting low toxicity.

As clarified in the carbon-based NPs section, graphene can either be coated onto the surface of the NPs or compose a composite with the NP to benefit the unique properties of the molecule. In the antibacterial application, this factor is significantly utilized with positive results. For example, a silver NP was decorated with the GO to form an antibacterial nanocomposite [501]. Along with the composite, GO and silver NPs were also used to compare their antibacterial activity against four types of bacteria: *E. coli*, *S. aureus*, *S. epidermidis*, and *C. albicans*. According to the cell viability results, the sole administration of GO and silver NPs showed similar activity with GO having a slightly better activity. On the other hand, the silver NP-GO composite showed significant activity with much higher results compared to sole administrations. As mentioned previously, silver NPs are known to cause disturbance and damage to the cellular membrane, which is behind its antibacterial activity. This was also tested to observe the effect of GO on the antibacterial activity by measuring the LDH levels in the medium. Similar to the significant increase in antibacterial activity, the composite also substantially increased the LDH levels. Finally, the composition slightly increased ROS production. The researchers discussed that, even though it is not clear, the aggregation of silver NPs during the action might be relieved by the GO, eventually leading to increased antibacterial activity. 

Similarly, the same composite with GO and silver NPs was synthesized, and their antibacterial activity and toxicity were evaluated [502]. Again, the decreased aggregation of silver NPs and the enhanced antibacterial activity of the composite were observed. One additional factor observed during this experiment was the toxicity comparison. The composite showed significantly lower toxicity (no toxicity was observed until the concentration reached 60 μL) against HEK293 cells, compared to silver NPs. Many similar studies currently exist in the literature, using GO to form composites to enhance the antibacterial activity of NPs. Here, we have discussed the use of GO with silver NPs since it was the most detailed antibacterial NP that we have encountered. However, the use of other types of NPs with GO is presented in Table 3.

#### 3.2.8. Carbon-Based NPs in Industrial Applications

As explained in the section on CBNPs, carbon black NPs have a limited role in biological applications due to their cytotoxicity and their emphasis on mechanical rather than biological features. Concurrently, CBNPs are also utilized in diverse fields, such as the design of electrochemical sensors, lithium batteries, and sodium batteries. Here, we briefly discuss a few studies to highlight the applications of CBNPs, with additional studies presented in Table 3.

CBNPs have been widely used in recent studies as reinforcing fillers for rubber-based materials. Thanks to the properties of carbon black, CBNPs enhance crucial properties such as tensile strength, tear resistance, and abrasion resistance [503]. For example, researchers investigated the effect of adding carbon black on the properties of various types of rubber, such as natural rubber (NR) and butadiene rubber, as well as their blends in varying compositions [504]. Their focus was mainly on how carbon black effects cure characteristics, density, hardness, and mechanical properties, including tensile strength, elongation at break, and tear strength. In conclusion, the findings revealed that the addition of carbon black reduced cure time, increased hardness and density, and significantly enhanced mechanical properties, particularly in those filled with NR70 compared to its counterparts.

However, an important drawback needs to be mentioned: the disruption of the carbon black’s reinforcement property at high temperatures. To exemplify, researchers treated CBNPs with various methods such as washing with water, alcohol, toluene, and a water–toluene emulsion and heated them to different temperatures to observe the changes in surface activity. Subsequently, it was stated that although the surface activity of CBNPs increased with an increase in temperature, a decrease in the effectiveness of rubber reinforcement was observed after 450 degrees Celsius [77]. 

Additionally, a study presented a cost-effective and efficient method using the catalytic properties of CBNPs to detect phosphate levels in water, which is essential for evaluating its quality and monitoring eutrophication levels [505]. Experimental results indicated significant sensitivity, with a low detection limit of 6 μM and excellent repeatability of the method, making it suitable for long-term monitoring of phosphate levels in various water sources.

As a final example, a study investigated the use of CBNPs in the development of lithium–sodium batteries [506]. Researchers specifically focused on modifying CBNPs by incorporating them with iron phosphide (FeP) NPs, aiming to enhance the battery performance. Findings revealed that this modification significantly improved sulfur redox kinetics, enabling long-term cycling at high rates. Furthermore, the uniform distribution of FeP NPs on carbon black maximized exposure to active sites, resulting in enhanced battery performance even at high sulfur loadings.

In conclusion, the advantageous features of CBNPs, such as their ability to serve as effective reinforcing agents and contribute to electrochemical applications, have enabled their applicability in the industrial area, despite their acknowledged limitations, including cytotoxicity.

Graphene is one of the leading materials in industrial applications, with high market value and wide range products supported by an extraordinary number of patents [507]. Both as an NP and material, it is involved in many materials for reinforcement due to its mechanical properties, CO_2_ conversion in composite form, metal and gas absorption, and so on [508]. A few examples that involve GO and other types of NPs will be briefly discussed to indicate the industrial application of graphene in the scope of this review.

To give an example, in a study similar to the application of carbon black NPs, GO NPs were hybridized with SiO_2_ NPs to reinforce the mechanical properties of nitrile rubber [509]. To determine the mechanical enhancement of the hybrid NP, nanocomposites were formed with nitrile rubber, including GO NPs, SiO_2_ NPs, and the hybrid NP. The researchers discussed the thermostability of graphene and its increased effect with the addition of O_2_-containing functional groups to graphene. As a result, the addition of SiO_2_ NPs increased the thermal stability and decreased the weight loss from 47 wt% to 26 wt% at 350 °C. The formed hybrid greatly increased tensile strength; however, the increase in elongation at break was lesser. Another type of material wherein GO NPs are used to enhance resistance is concrete. An experiment showed the enhanced blast load resistance of steel fiber-reinforced concrete with GO NPs [510]. In the first experiment, certain amounts of GO NP slightly decreased compressive strength. The researchers indicated that the addition of GO NPs increased the surface area and caused distress in obtaining compaction. Conversely, they found that the optimum amount of GO NP (0.025%) increased flexural strength by 8.22%, as the bonding between the fiber and matrix strengthened. Subsequently, blast loading tests were performed, and the addition of GO NPs showed better damage patterns as a result. Finally, GO NPs decreased permanent deflection due to the bond between the NPs and steel fibers. Many other similar types of research use GO and GO NPs as reinforcing materials due to their rich physical properties. GO is either utilized with other types of NPs to combine their properties, especially physical and mechanical or is solely used in diverse materials.

### 3.3. Organic NP Applications

Organic NPs have a particular place in NP application, as they possess significant biocompatibility, ease of functionalization, and low toxicity. Their organic composition enables them to interact easily with biological systems, making them suitable materials for a broad range of applications, such as wound healing, diabetes research, drug delivery, and food packaging (Table 4).

#### 3.3.1. Organic NPs in Wound Healing Applications

Encapsulating hydrophilic and hydrophobic materials with organic NPs becomes an advantageous approach for wound healing studies as they can modulate inflammation, promote angiogenesis, and upregulate growth factors, which are crucial for accelerating the tissue repair process and thereby the rate of wound healing. For example, PLGA NPs loaded with LL37, a host defense peptide, were synthesized to evaluate their potential in promoting wound healing [511]. Once the NPs were engineered to optimize efficient LL37 encapsulation, *in vivo* experiments conducted on murine models demonstrated that these NPs significantly accelerated wound closure, upregulated IL-6 and VEGFa expression, enhanced epithelialization, and improved angiogenesis compared to control groups. Additionally, sustained release of LL37 from the NPs was observed to effectively modulate the inflammatory response, which is considered crucial for an efficient healing process.

Also, another study was conducted to explore the effectiveness of curcumin-loaded liposomes on wound healing [512]. The researchers formulated the liposomes to improve the bioavailability and solubility of curcumin, known for its significant anti-inflammatory properties. After successfully optimizing the liposome formulation, various tests, including *in vitro* drug release studies, encapsulation efficiency measurements, and *in vivo* experiments, were applied to assess the performance of the curcumin-loaded liposomes. Findings indicated that curcumin was released continuously at the wound site, significantly promoting the healing process, as evidenced by improved wound closure rates and a notable decrease in inflammation.

Researchers synthesized vaccarin–chitosan NPs (VAC-NPs) to investigate their potential in the wound healing process [513]. The synthesis phase employed the ionic gelation method, which involves crosslinking chitosan with tripolyphosphate ions to ensure effective encapsulation of vaccarin. Subsequent *in vivo* experiments conducted on rat models demonstrated that VAC-NPs significantly promoted wound closure and facilitated the sustained release of vaccarin, thereby effectively moderating the inflammatory response. It was also stated that this mechanism might have arisen from the upregulation of IL-1β and PDGF-BB, well-known factors in tissue repair and regeneration, further promoting angiogenesis and efficiently accelerating wound healing.

In conclusion, the current literature features various studies with similar findings utilizing LBNPs, especially liposomes, for wound healing applications. To sum up, LBNPs’ advantageous characteristics, such as being able to encapsulate both hydrophilic and hydrophobic molecules effectively, not only establish their significance but also emphasize their further potential in this field. 

#### 3.3.2. Organic NPs in Diabetic Research

Here, we explore the involvement of fullerenes in diabetic research, highlighting a few studies that present their molecular structure and high antioxidant capacity. In this context, most organic NPs are included in diabetic research for their mucoadhesive and antibacterial traits, rather than their antioxidant activity. As a result, alginate NPs have a substantial place in diabetic research. Since both chitosan and alginate possess mucoadhesive characteristics, many diabetic-based NP studies include these two polymers in their experiments. The mucoadhesive trait is crucial in site-specific drug delivery, such as in the gastrointestinal regions and respiratory and reproductive systems, as many organs in these regions possess mucous membranes and are susceptible to mucus-related diseases [514].

Based on this, NPs with mucoadhesive features have the advantage of increased drug delivery efficiency in these regions. Using mucoadhesive polymers in NP synthesis, primarily alginate, and chitosan, covers this advantage, and their non-toxicity reputation strengthens it. The utilization of these polymers can increase the duration of drug exposure in these regions, improve the durability of the drug against the removal effects, and decrease the cost and required dose for administration [515,516]. As an example, chitosan and alginate shell NPs were synthesized to orally deliver a polyphenol, naringenin, to a diabetic animal model [517]. The synthesized NPs were characterized in terms of encapsulation efficiency, drug release efficiency based on pH levels, and mucoadhesion to determine intestinal mucosal attachment. Along with the approximately 91% encapsulation efficiency, the NP managed to release 90% of the drug at a pH level of 7.4 in the animal model. Later, an ex vivo mucoadhesive test was performed with a rat intestinal lumen. The NPs showed significant binding strength to the intestinal lumen, even after the washing processes. There was no trace of toxicity, which is expected given the non-toxicity and biodegradability of polymeric NPs. For 19 days, 50 mg/kg alginate–chitosan NPs loaded with naringenin were administered to the diabetic rats, along with a control group. Blood glucose levels were determined, and by day 30, the NP-administered rat group showed almost the same level of blood glucose as the control non-diabetic group.

Independent of the mucoadhesive trait, alginate and chitosan are still utilized in NP applications for diabetic research. For example, an interesting combination of NPs was synthesized for the healing of diabetic wounds [518]. Two NPs, silver and calcium alginate, were prepared—while chitosan served as a base matrix (to enhance biodegradability) during the synthesis—to test the antimicrobial (from the silver ions) and hemostatic (from the alginate) effects. Two Gram-negative (*Pseudomonas aeruginosa*, *E. coli*) and two Gram-positive (*B. subtilis*, *S. aureus*) bacteria were tested for antibacterial activity. Following the successful antibacterial activity of the AgNPs, an *in vivo* wound healing experiment was performed on diabetic rats. The wound reduction reached up to 99% in the blood-mixed NP combination, and 83% in NP administration, indicating their significant potential in tissue repair. Other types of polymers have also been shown to be effective against diabetic wounds in NP forms, such as polycaprolactone [519] and PLGA [520].

**Table 4:** Applications of organic NPs.

Type of Organic NP	Application Area	Highlighted Results	References
Chitosan	Wound healing	- Hybrid NP with alginate to heal both diabetic and non-diabetic wounds (*in vivo*) - Increasing the healing process of diabetic wounds and carrying agents (*in vivo*)	[521,522,523]
Chitosan	Diabetes	- Polydatin-loaded chitosan NPs decrease the progress of diabetic nephropathy and liver damage (*in vivo*) - Protection of cardiac cell damage by antioxidant and anti-apoptotic activity (*in vivo*)	[524,525,526]
Chitosan	Drug delivery	- Delivery of type-2 diabetic drug with up to fourfold enhancement (*in vivo*) - Delivery and enhanced release of rosuvastatin with hydrogel film - Multiple types of antibiotic drug delivery - Potential drug delivery candidate for ophthalmic drugs	[527,528,529,530]
Chitosan	Food preservation	- Biopreservation of shrimp (unaffected color and texture) with antibacterial activity - Preservation of pork by preventing biofilm formation and antibacterial activity, with mandarin essential oil encapsulated. - Effective antibacterial activity against foodborne bacteria with encapsulation of multiple essential oils - Increased shelf life of bell pepper with antibiofilm and antioxidant activity	[531,532,533,534]
Chitosan	Food packaging	- Quercetin-encapsulated chitosan NP exhibited UV barrier, antioxidant, antibacterial, and improved mechanical properties in food simulant solutions - Chitosan NP bilayer film for oily food packaging showed significant antioxidant and antibacterial activity, along with oil resistance and increased optical properties	[535,536]
Alginate	Diabetes	- Mangiferin-loaded alginate NPs show potential in oral delivery for diabetes-mediated hyperlipidemia (*in vivo*) - Glucose oxidase-loaded alginate NPs utilized for enhanced glucose-based insulin delivery in terms of glucose response and biocompatibility (*in vivo*)	[537,538]
Alginate	Drug delivery	- Co-synthesis of alginate NP with chitosan significantly increased the antibacterial activity of oregano oil against multiple strains - Enhanced stability, skin delivery, and sustained release profile of protein hydrolysate from *Acheta domesticus* - *Coccinia grandis* L. extract encapsulation exhibited enhanced loading capacity, encapsulation efficiency, desired release and preferred structural traits for antidiabetic drug system	[539,540,541]
Alginate	Food preservation	- Coating of alginate NP into strawberries, guava, and pumpkin seeds exhibited protection by antimicrobial and antioxidant activity - Essential oil-encapsulated alginate NPs exhibited increased antibacterial activity and shelf life of shrimp in storage	[542,543]
PLGA	Drug delivery	- PLGA-NPs show efficient ROS-sensitive co-delivery of drugs for colon cancer treatment - PLGA-NPs show potential as a drug delivery system for inner ear diseases with positive drug release profile (*in vivo*, *in vitro*) - Enhanced docetaxel delivery on multiple human cancer lines with extended blood circulation in modified form (*in vivo*, *in vitro*) - Enhancement in terpene delivery in terms of drug release, stability, and bioavailability.	[544,545,546,547]
PLGA	Tissue engineering	- Aspirin-loaded PLGA NPs on curcumin membrane exhibited osteogenic and antibacterial activity (*in vivo*, *in vitro*) - PLGA NPs utilized in the development of alginate-based bio-ink for bone tissue engineering - IGF-1-loaded PLGA NPs were included in scaffold fabrication for enhanced cartilage tissue engineering with increasing IGF-1 release	[548,549,550]
PLGA	Wound healing	- Alkaloid-loaded PLGA-NPs, coated with chitosan, successfully maintain infected wounds by antibacterial and wound healing activities - Heparin-loaded PLGA NPs exhibited significant skin regeneration potential	[551,552]
PLGA	Diabetes	- PLGA NPs enhance the oral delivery of insulin by showing high biocompatibility and bioavailability and regulating blood glucose levels (*in vivo*, *in vitro*) - PLGA NPs, conjugated with heparin sulfate, enhanced insulin protection and permeability - Modified PLGA NPs shows great potential for treating diabetic retinopathy by successful delivery and distribution of pioglitazone (*in vivo*, *in vitro*)	[553,554,555]
PLGA	Imaging	- A tumor-targeting PLGA NP was used to deliver and monitor the treatment of ovarian cancer resistance by allowing for ultrasound and magnetic resonance imaging (*in vivo*) - Functionalized chitosan–PLGA NP was developed for antitumor activity and imagining in brain cancer cells (*in vivo*, *in vitro*)	[556,557]
Liposome	Drug delivery	- Sustained delivery of resveratrol for protecting retina from blue light - A drug delivery system including alginate–chitosan hydrogel and tetramethylpyrazine-loaded liposomes enhanced the antibacterial, antioxidant, and anti-inflammatory properties of the drug for atopic dermatitis treatment (*in vivo*, *in vitro*) - Functionalized liposomes enhanced the delivery honokiol for breast cancer inhibition with increased distribution, cytotoxicity, and anti-migration (*in vivo*, *in vitro*)	[558,559,560]
Liposome	Cosmetics	- Recombinant human growth hormone-loaded liposomal formulation efficiently prevented UVB skin damage and showed potential anti-wrinkle and collagen loss prevention activity (*in vivo*, *in vitro*) - *Caryocar brasiliense* fruit pulp oil was encapsulated into freeze-dried liposomes and showed enhanced skin hydration, protection, and improved skin conditions	[561,562]
Liposome	Wound healing	- Shikonin-encapsulated liposome exhibited significant wound healing by preventing infection, inflammation with decent stability, dispersion, and repair promotion (*in vivo*, *in vitro*) - Similarly, taxifolin-encapsulated liposome possessed wound healing activity with interfering signaling pathways in diabetic mice (*in vivo*) - Encapsulation of SB431542 showed significant wound healing activity with minimal amounts of scar formation in rat model (*in vitro*, *in vivo*)	[563,564,565]
Liposome	Food preservation	- Essential oil encapsulated liposome, modified with alginate and chitosan, exhibited antiseptic activity on chilled pork - Encapsulation of *Litsea cubeba* essential oil enhanced preservation of salmon by increased antibacterial activity and decreased salmon oxidation - Polyvinyl alcohol–chitosan-loaded baicalin liposomes successfully maintained nutrient value of mushrooms, prevented weight loss, and possessed significant antibacterial activity	[566,567,568]
Liposome	Gene delivery	- A modified liposome was utilized to deliver a therapeutic gene, acetylcholinesterase, for liver cancer; liver cancer growth was significantly decreased with the enhanced targeting and transfection (*in vivo*, *in vitro*) - Brain-targeted delivery of ApoE2 encoding plasmid DNA was performed with modified liposomes for Alzheimer’s disease (*in vivo*, *in vitro*)	[569,570]
Solid lipid	Food preservation	- SLNs showed a potential to be an alternative as a coating material for strawberry preservation in certain concentrations - Encapsulation of curcumin showed significant antibacterial activity against foodborne pathogens, indicating the potential preservation of hamburger patty - Curcumin-loaded SLNs exhibited significant photodynamic inactivation and antibacterial activity for carrot juice preservation	[571,572,573]
Solid lipid	Drug delivery	- Enhanced delivery, bioavailability (up to 12-fold), and stability in curcumin (*in vivo*, *in vitro*) - Morin hydrate-loaded SLNs enhanced the bioavailability and anticancer activity (three times higher) - Dual loading of curcumin and dexanabinol into SLNs exhibited great potential to treat major depressive disorder by positively influencing mRNA, protein, and dopamine expressions (*in vivo*)	[574,575,576,577]
Solid lipid	Cancer	- Gemcitabine-loaded SLNs possessed enhanced delivery and anticancer activity against pancreatic cancer cells - Functionalized SLNs show great potential in ovarian cancer targeting coupled with higher cytotoxicity and effective internalization - SLNs were utilized to enhance 5-fluorouracil’s activity in colorectal cancer (*in vivo*, *in vitro*)	[578,579,580]
Solid lipid	Cosmetics	- Synthesized SLNs from Otoba wax possessed great potential in hair cosmetic formulations - Prunus persica (L.) leaf-loaded SLNs increased the efficiency of skin delivery and showed potential for anti-wrinkle applications (*in vivo*, *in vitro*) - SLNs were used as a carrier of fucoxanthin for its UV protection, which led to an increased effect. Enhanced properties by SLNs indicated its potential to be used in sunscreen products - SLNs successfully delivered multiple lipophilic compounds by increasing their bioavailability for skin cell aging	[581,582,583,584]
Nanostructured lipid carriers	Drug delivery	- NLCs enhanced drug release of ibrutinib and skin absorption for melanoma treatment - Functionalized NLCs loaded with isoniazid possessed potential in pulmonary tuberculosis by increased bioavailability and therapeutic efficiency (*in vivo*, *in vitro*) - NLCs improved the uptake, stability, release, and therapeutic effect of kaempferol on glioblastoma multiforme cells - NLCs showed stable, mucoadhesive, and non-toxic traits in the delivery of Lf for keratoconus treatment (*in vivo*, *in vitro*) - Paclitaxel-loaded NLCs possessed high entrapment efficiency, stability, and burst drug release for retinoblastoma	[585,586,587,588,589]
Nanostructured lipid carriers	Cancer	- Increased efficiency and drug release of propolis and umbelliprenin in breast cancers (*in vivo*, *in vitro*) - Increased drug release profile and cytotoxicity of gefitinib for lung cancer treatment - Dual encapsulation of quercetin and resveratrol significantly improved accumulation, cytotoxicity, and anti-metastatic activity for treating skin cancer - Multifunctionalized dihydroartemisinin-encapsulated NLCs possess the potential for glioma with great release profile, stability, and encapsulation	[590,591,592,593,594]
Nanostructured lipid carriers	Cosmetics	- Reduction in hair wick frizz and increased hair brightness - α-tocopherol-loaded NLC gel possessed suitability to be applied in sunscreens without pH-based irritation - Coenzyme Q10-loaded NLCs showed significant antioxidant activity as a potential material for skincare products (*in vivo*, *in vitro*)	[595,596,597]
Nanostructured lipid carriers	Food preservation	- Thymol-encapsulated alginate-coated NLCs showed significant antimicrobial and antioxidant activity in the preservation of chicken meat - Gelatin-coated NLCs, encapsulated with *Salvia officinalis* extract, exhibited great antibacterial and antioxidant activity in the preservation of beef burger - *Thymus vulgaris* essential oil-encapsulated NLCs, coated with alginate, significantly expanded the shelf life of tangerine fruit by high antioxidant capacity and phenol content, with the lowest weight loss - Thymol-encapsulated alginate-coated NLCs enhanced the postharvest quality of carrots and increased their shelf life (still at day 30)	[598,599,600,601]
Cyclodextrin	Cancer	- P-gp efflux pumps could be inhibited by CD NPs and tests for cytotoxicity revealed that the NPs had no negative effects.	[136,138]
Starch	Drug delivery	- Usage of starch NPs provided fewer side effects, increased solubility and bioavailability, improved therapeutic control, increased cellular absorption into A549 cells, and increased cytotoxicity. - The produced NPs’ pH-dependent properties were demonstrated by the outcomes of *in vitro* drug release investigations. The produced NPs’ biocompatibility with L929 fibroblast cell lines was demonstrated by an *in vitro* cytotoxicity test.	[163,602]
Starch	Antimicrobial agent	- The compound’s retention capacity ranged from 41.5 to 90 mg g^−1^, indicating its potential as an antibacterial agent in food systems	[603]
Lactoferrin	Drug delivery	- The drug payload was delivered into the cell via the NPs, which also showed a second function by preventing virus invasion. - Disulfiram–Lf NP therapy showed exceptional therapeutic benefits for sepsis brought on by lipopolysaccharide (LPS).	[604,605]
Lactoferrin	Antimicrobial activity	- Binary complexes (Lf–gellan) and ternary complexes (Lf–chitosan–gellan) were investigated. The antibacterial activity of nanoparticles against *S. aureus* was evaluated in relation to nanoparticle size, charge density, and shape, and was compared with that of pure biopolymers.	[606]

#### 3.3.3. Organic NPs in Drug Delivery Systems

The non-toxic and highly biodegradable traits of organic NPs are extensively utilized in drug delivery applications. In fact, all of the subclasses of organic NPs offer several advantages in drug delivery. The most featured characteristics include high stability and storage capability, specific targeting and accumulation, enhanced circulation, controlled drug release, and so on [143]. Owing to these features, many organic materials and their NP forms have inevitable potential in this area.

Lf possesses multiple traits that enhance its application as an NP in drug delivery. One of them is the ability of Lf to cross the blood–brain barrier (BBB) via receptor-mediated transcytosis, and the existence of Lf receptors in certain neuronal and endothelial cells in the brain regions [297]. To come up with an efficient therapy for brain-related diseases, especially neurodegenerative diseases, overcoming the BBB passage is the most important feature in drug delivery [607]. This is why Lf is considered an efficient material in drug delivery systems to the brain regions, coated onto the surface of NPs. 

As an example, curcumin and Lf NPs were synthesized for nose-to-brain delivery to generate neuroprotective activity both *in vivo* and *in vitro* [608]. The *in vitro* experiments were conducted on PC12 cells, where NP-mediated curcumin showed faster drug release, higher drug uptake, and higher protectivity capacity compared to the free form of curcumin. Conversely, an *in vivo* experiment showed that curcumin had an increased half-life and improved bioavailability during delivery. A similar approach was employed with NLC loaded with riluzole and functionalized with Lf to enable drug delivery into brain regions [609]. The significant loading capacity, stabilization, and highly degradable, non-toxic nature of NLC are combined with LF to address both the BBB issue and improve drug delivery efficiency. An *in vitro* experiment performed with two cell lines, NSC-34 and hCMEC/D3, showed promising results that indicate the potential of both NLC and Lf, with nearly 98% encapsulation efficiency. Lastly, a previous study demonstrated the *in vivo* nose-to-brain delivery of NAP peptide by PEG-modified Lf NPs for Alzheimer’s disease [610]. The results not only showed a dose-dependent neuroprotective relationship but also increased the scores of behavioral tests on mice.

Drug delivery applications of organic NPs are not limited to brain regions; different types are preferred for delivering drugs to certain regions. Chitosan NPs are widely used in cancer and tumor studies due to their mucoadhesive, anticancer, and antioxidant properties [611]. For instance, an *in vitro* study utilized gefitinib-loaded chitosan NPs on A549 cells for lung cancer [612]. As mentioned in the diabetes section, the mucoadhesive trait is crucial in the respiratory system, making chitosan a compatible choice for this targeted area. Administration of the drug with the chitosan NP positively altered the following results: the apoptosis assay showed a significant increase in apoptosis rate compared to sole drug and control groups, an almost twofold increase in the drug uptake with NP modification, and an increase in cytotoxicity followed by the uptake. A similar study was conducted in *in vivo* and *in vitro* conditions for breast cancer [613]. Doxorubicin-loaded chitosan NPs, with cinnamaldehyde, were tested in MCF-7 cells and showed a pH-dependent increase in toxicity against cancer cells. Subsequently, an *in vivo* experiment showed increased doxorubicin accumulation and reduced tumor growth when administered with the NP form.

CDs have the capacity to deliver desirable compounds to biological targets, enhancing their solubility, stability, and supramolecular functionalization characteristics. However, folate receptors or epidermal growth factor receptors are typically the targets currently being used. To improve the accuracy of tumor identification, CD NP functionalization should be tailored for additional cancer biomarkers and receptors [614]. In 2020, Yusheng et al. developed an NP based on β-cyclodextrin (βCD) to investigate its anticancer properties *in vitro*. Cell-penetrating peptides (CPPs) enhance the adsorption of the cancer medicine that the target tumor cells take up by increasing the NPs’ delivery to the cells [138].

LBNPs have unique advantages in drug delivery applications, particularly in enhancing aqueous solubility and protecting drugs from oxidation and degradation [615]. As a result, they are predominantly used and demonstrate high efficiency in delivery systems.

As an example, a study explored the therapeutic potential of *Aphanamixis polystachya* leaf extract, known for its medicinal properties, encapsulated in liposomes. Researchers formulated stable liposomes and analyzed their characteristics using various techniques such as TEM and FT-IR. *In vivo* studies using mouse models subsequently demonstrated notable improvements in locomotor activity, memory, and anti-inflammatory response with the liposomal formulation compared to the extract alone, emphasizing the potential of liposomal drug delivery in enhancing the therapeutic efficiency of *Aphanamixis polystachya* [616].

On the other hand, SLNs, a subset of LBNPs, also show promise in drug delivery applications. For instance, a study described the development and characterization of SLNs conjugated with transferrin for the delivery of tamoxifen citrate, a widely used therapeutic agent for breast cancer therapy. NPs engineered to optimize parameters such as particle size, drug encapsulation, and stability underwent comprehensive *in vitro* experiments conducted on breast cancer cell lines. The results indicated effective inhibition of cell proliferation, demonstrating the potential of SLNs as drug delivery systems for the treatment of breast cancer [617].

Finally, considering the NLCs, researchers aimed to overcome the poor oral bioavailability of astaxanthin (AST), a lipophilic compound known for its antioxidant, anti-inflammatory, and neuroprotective properties in the treatment of Alzheimer’s disease. The aim of encapsulating AST in NLCs was to enhance its delivery efficiency to the brain via the nasal route. Experiments conducted in rat models demonstrated significant reductions in oxidative stress, amyloidogenic pathway activation, neuroinflammation, and apoptosis following intranasal administration of the optimized AST-loaded NLCs. Furthermore, the NLCs improved cholinergic neurotransmission in AD-like rats when compared to AST solution [618].

Moreover, NLCs possess mucoadhesive properties, similar to the polymers mentioned earlier, which are considered crucial for drug delivery applications. In line with this, a study was carried out to utilize the mucoadhesive properties of NLCs in ocular drug delivery, particularly focusing on targeting regions near or related to the eye [619]. Specifically, researchers developed Lf-loaded NLCs aiming to create stable formulations with desirable physicochemical properties. Subsequently, the resulting NLCs underwent comprehensive evaluation, including assessments of particle size, size distribution, surface charge, morphology, encapsulation efficiency, loading capacity, stability, cytotoxicity, *in vitro* release, and ocular surface retention. These analyses revealed uniform particle size, high encapsulation efficiency, and sustained drug release characteristics, consistent with mucoadhesive traits of NLCs. Overall, considering the importance of mucoadhesive traits in ocular drug delivery, these findings highlight the potential applicability of mucoadhesive-based formulations using NLCs for optimized and sustained delivery of drugs to the eye [588].

#### 3.3.4. Organic NPs in Food Packaging and Food Preservation Applications

The utilization of organic NPs is widespread across many subclasses, particularly in polymeric and LB NPs. Given that many of the highlighted traits are commonly shared among these groups, the current literature includes a significant amount of research on the use of organic NPs in food packaging and preservation.

Chitosan is commonly used in food packaging studies, especially for its antimicrobial properties [620]. For example, a recent study synthesized chitosan NPs, along with alumina NPs, and combined them with grape extract to create a film for food packaging [621]. The combined film exhibited significant biodegradability, antimicrobial activity, and pH sensitivity. Similarly, a film generated from carboxymethyl cellulose and starch was combined with chitosan NPs for food packaging [622]. The synthesized nanocomposite film was tested for multiple properties and on chicken meat. This not only increased the shelf life of the chicken meat but also improved UV blocking and antioxidant capacity, along with decreasing permeability. 

Other types of polymers are also used for similar purposes in food packaging. As mentioned, PLA possesses high biodegradability and is transformed into CO_2_ as an end product during degradation. Similar advantageous features are present in most of the polymers used in NPs, which is why they are preferred in this area. For example, a PLA NP containing green tea extracts was designed for food packaging [623]. The researchers aimed to generate a highly antioxidant active film to protect high-fat food products from lipid oxidation, and they designed PLA NPs to achieve the desired antioxidant levels. 

These polymers are not solely used in food packaging, especially when considering PLA. In terms of NP applications, it is sometimes preferable to use the polymer with the inorganic NPs rather than using the polymer itself as an NP. Since inorganic NPs can exhibit significant antimicrobial activity, certain inorganic NPs enhance the properties of PLA and the designed NP in food packaging applications [624]. For instance, a nanocomposite PLA film with functionalized silica NPs was designed to refer to a case study [625]. The characterization and analysis of the NP properties were compared among sole PLA, PLA NPs, and PLA–silica NPs. As a result, lactic acid-functionalized silica NPs with PLA showed improved properties and potential as a packaging material. Similar polymer–metal hybrid NP applications also exist for other types of polymers, such as chitosan [620].

These traits enable the application of organic NPs in food preservation studies. Sharing similarities with food packaging in terms of desired outcomes, many studies aim to extend the shelf life of certain products from the same perspective. As an example, a recent study synthesized SLNs, coated with alginate, and encapsulated an essential oil, Zataria multiflora, to increase the antimicrobial activity and shelf life of chicken meat [626]. Most essential oils possess disadvantages such as hydrophobicity and sensitivity in administration, making encapsulation essential for efficient delivery and activity [627]. Since SLNs, and their other lipid-based derivatives, are preferred for the delivery of hydrophobic and hydrophilic compounds, using these kinds of nanocarriers in related research is a strategic approach. The results showed that SLN encapsulation enhanced the antioxidant and antibacterial activities, eventually increasing the storage time of the chicken meat.

A similar goal was achieved using Lf and chitosan NPs, linked with tripolyphosphate, to extend the shelf life of strawberries [628]. Due to their proven antibacterial activity, chitosan and Lf were the preferred materials for the NP application. Lf and chitosan NPs were tested solitarily and together in combined form. First, their antibacterial efficiency was tested, showing a significant decrease in their minimum inhibitory concentrations against *S. aureus* when they were used together. Later, the weight loss of the strawberries was examined, and it was the lowest in the combination of two NPs in the application at the 144th hour. The same researchers conducted a similar experiment on strawberries. Lf, chitosan, and gellan NPs were synthesized and applied as a complex in strawberry preservation [606]. Similar to the previous research, *S. aureus* was used for antibacterial activity comparison, which was higher in combined application than in their pure forms.

The application of LBNPs is extremely wide-ranging—due to the many properties that have been discussed—such as in improving water vapor barriers, decreasing decay rates, preventing pathogen growth, maintaining food quality, and many more [629]. It is not possible to mention all the traits of LBNPs in food packaging and preservation. Therefore, to cover its application briefly, a few examples will be discussed. Additional applications are already mentioned in Table 4. 

As an example, SLNs are combined with *Mentha × piperita* L. essential oil for edible coatings to preserve strawberries [630]. The effect of the protective coating on strawberries was tested against various factors. First, it was observed that the coating significantly reduced the weight loss of the strawberries in an observation of 20 days. The texture and the color of the strawberries were greatly preserved compared to the control group as well. Finally, the antioxidant and antimicrobial activity were the highest and protected the strawberries against *Rhizopus stolonifer* for 20 days. A similar application was shown with cinnamaldehyde-added SLNs in strawberries in a recent study [631]. Similar factors, such as spoilage, weight loss, texture, and color, were tested and showed positive results similar to the previous study. 

Liposomes are also extensively involved in food packaging and preservation studies, especially with their highlighted properties in capsulation molecules. An edible coating was created with liposomal chitosan by loading thyme essential oil to preserve Karish cheese [632,633]. To test the antibacterial activity, the change in total mesophilic bacteria was calculated, and preservation of the cheese was observed. The uncoated and chitosan-coated cheese started to show visible signs of mold and yeast growth in the second week. In contrast, the liposome-coated cheese did not show any visible changes at the end of week 4. Bacteria counts showed that the liposome coating significantly reduced the bacterial count over the entire 4-week storage period. Psychrotrophic bacteria and yeast counts were also the lowest in the liposome-coated samples. 

Similarly, it was demonstrated that liposome-coated citral showed promising results in extending the shelf life of Shatangju mandarin [633]. An antimicrobial assay was performed to demonstrate the antimicrobial activity of citral-encapsulated liposomes. The antimicrobial activity was significantly observed against *E. coli*, *S. aureus*, *B. subtilis*, and *Penicillium italicum.* When the citral-loaded liposomes were tested on mandarin, they showed the lowest decay at 56.67%, compared to the control group at 100%, and the free citral group at 97.78%, indicating the role of liposomes in achieving the desired results.

#### 3.3.5. Organic NPs in Cosmetics

Recently, lipid-based NPs have drawn significant attention in cosmetics, thanks to their non-toxic and biodegradable nature. These characteristics not only ensure safety for users and the environment but also improve the effectiveness of cosmetic formulations by enabling targeted delivery and stability of materials, thereby enlarging their applicability in skincare and beauty products [634]. For example, researchers investigated the efficiency of day and night creams containing *E. guineensis* (also known as red palm) fruit extract-loaded SLNs to improve various aspects of skin health. Through a 30-day application process involving 68 female volunteers aged 25–50 years, the creams demonstrated significant benefits. In particular, they effectively increased water accumulation in the epidermis, reduced water loss from the skin, enhanced skin elasticity, and did not cause any irritation. Furthermore, both creams led to a reduction in melanin content, resulting in brighter and clearer skin [635]. In another study, the aim was to achieve skin retention and minimize the side effects commonly associated with traditional treatments. Researchers evaluated the efficiency of a liposomal cream containing doxepin, an antidepressant exhibiting anti-inflammatory properties, for topical delivery. Through various analyses, including stability assessments, ex vivo permeation studies, and characterization of liposomes, findings revealed that the liposomal formulation demonstrated improved skin penetration and retention of doxepin compared to plain cream formulations [482]. In conclusion, these examples indicate the potential of novel formulations using NPs in cosmetics and skincare, emphasizing the current progress in improving skin health through innovative applications. 

## 4. Toxicity 

**Figure 7:**
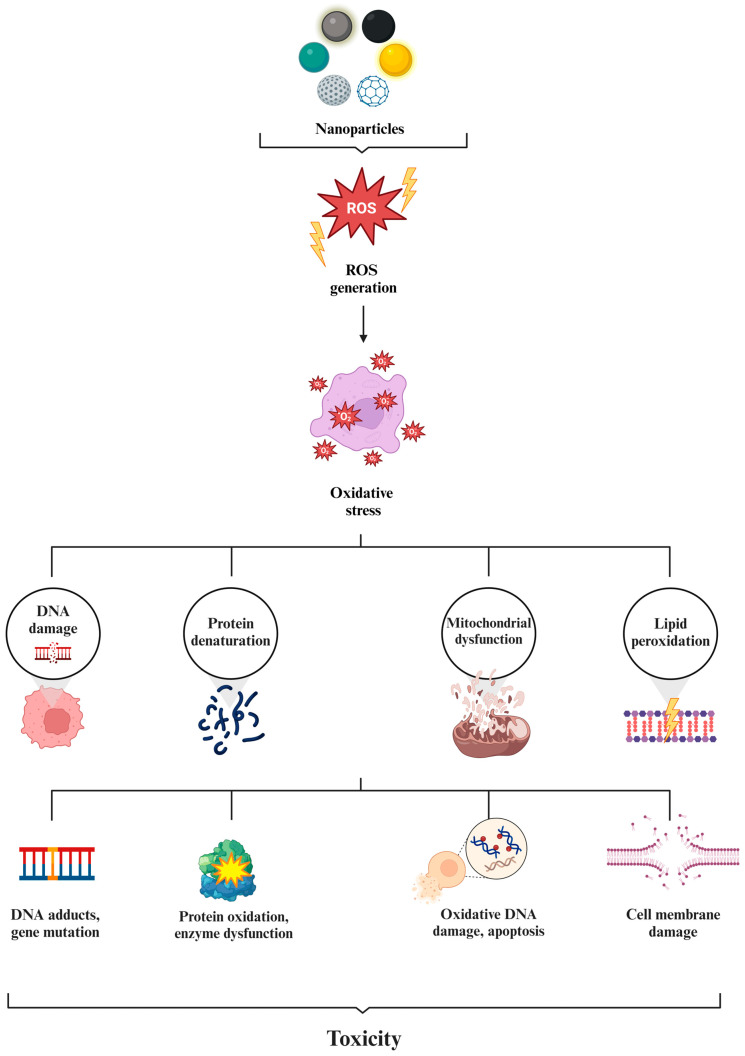
Representation scheme of toxicity mechanism of NPs [637].

Toxicity is an essential factor to consider since the application of NPs includes tissue repairing, drug delivery, food packaging, and similar derivative areas. The base material affects the toxicity, and its mechanism is similar to the area of applications. The toxicity of an NP is dependent on the administration concentration, stability, and bioavailability in the biological system and the tendency to accumulate in an organ or tissue [636]. Each of these factors varies based on the material and type of the NP, alongside the properties of size and surface [637]. Regardless of the material, NPs can exhibit toxicity by ROS synthesis (Figure 7).

Inorganic NPs, such as metallic NPs, are the most commonly commercially produced nanomaterials. They can be easily incorporated with other substances and employed as therapeutic agents in anticancer, antioxidant, and antibacterial applications. However, their increased exposure may lead to adverse toxic effects, including oxidative stress, inflammation, and genotoxicity [16].

Silver NPs, widely investigated for their potent antimicrobial properties, are highly valued across diverse fields such as medicine, biotechnology, and environmental science. However, concerns about their toxicity to human health and the environment have emerged. The toxicity of silver NPs is influenced by factors including particle size, shape, surface charge, coating, and concentration. Also, their smaller size and the release of silver ions contribute to increased reactivity, potentially causing cellular damage, oxidative stress, and inflammation [638].

A study investigated the toxicity of silver NPs, using zebrafish embryos as a model organism. Results indicated that exposure to silver NPs led to dose-dependent adverse effects, including increased mortality rates, delayed hatching, pericardial edema, cardiac arrhythmia, and developmental abnormalities such as twisted notochords in zebrafish embryos [639].

Another study assessed the toxicological effects of silver NPs on male rat organs, especially the liver, kidney, and heart, following subdermal exposure at varying concentrations over 14 and 28 days. Results revealed significant oxidative stress in these tissues, indicated by elevated levels of lipid peroxidation products such as malondialdehyde. The study also found notable reductions in antioxidant defenses, both enzymatic (CAT, SOD) and non-enzymatic (GSH). Moreover, liver enzyme activities (ALT, AST, and ALP) showed marked elevation, suggesting hepatotoxicity, while kidney function was impaired due to increased urea and creatinine levels and renal damage. To add more, cardiac tissues exhibited oxidative damage and inflammatory responses following exposure to silver NPs [640]. Concerning another type of iNP, researchers investigated the *in vivo* toxicity of gold NPs in mice models, focusing on size-dependent effects ranging from 3 to 100 nm. Findings demonstrated that gold NPs between 8 and 37 nm induced toxicity, including loss of appetite, fatigue, weight loss, and structural abnormalities in major organs like the lungs, liver, and spleen [641]. In a study, researchers investigated the dose-dependent *in vivo* toxicity of silver NPs in rat models [642]. Based on the findings, silver NPs in doses less than 10 mg/kg are considered safe for biomedical applications. However, higher doses, particularly 20 and 40 mg/kg, exhibited increased ROS levels compared to other groups, thereby leading to toxicity. Similarly, another research group evaluated the toxicity of TiO_2_ NPs by specifically focusing on dose and particle size configurations. *In vivo* experiments on mice models demonstrated that TiO_2_ NPs showed significant toxic effects, including oxidative stress, genetic alteration, and organ damage, particularly with higher doses and smaller particles [643]. Therefore, when conducting *in vivo* experiments utilizing inorganic NPs, the correlation between dosage size and toxicity rates must be considered. 

Yet, various surface modifications can potentially affect the toxicological outcomes of inorganic NPs. For example, researchers utilized ZnO NPs to overcome toxicity through surface coating. By modifying the surface chemistry of ZnO NPs with various surfactants, including PEG, cetyltrimethylammonium bromide, and sodium dodecyl sulfate, they aimed to mitigate toxicological effects. Through *in vitro* studies, it was revealed that pure ZnO NPs induced cytotoxicity, apoptosis, DNA fragmentation, and mitochondrial dysfunction, while surfactant-coated counterparts exhibited a reduction in these toxicity mechanisms [644].

Recently, IONPs have attracted considerable interest due to their unique magnetic properties and potential applications in various fields, including biomedical and environmental sectors. However, these properties also raise concerns regarding their potential toxicity when in contact with biological systems [645]. Hence, understanding the toxicological profile of IONPs is crucial for their safe utilization in advanced technologies. In this manner, researchers investigated the cytotoxic effects of various NPs, including IONPs, on human periodontal ligament fibroblasts and mouse dermal fibroblasts. They employed various assays including MTT, LDH release, ROS measurement, and cellular impedance to evaluate cell viability, oxidative stress, and proliferation dynamics under NP exposure. Results indicated that IONPs induced significant cytotoxic responses in both cell types, characterized by elevated ROS levels, impaired cellular impedance, and altered cell morphology [646]. Moreover, another study investigated the toxic effects of IONPS on the kidneys, liver, and brain of male mice. The NPs were administered orally at concentrations of 6%, 8%, 10%, and 12% every 48 h for 60 days. Results revealed that although the lower doses (6% and 8%) were not significantly toxic, the higher doses (10% and 12%) led to blood congestion, inflammation in the kidneys, liver cell enlargement, and brain congestion, indicating potential toxicity at elevated concentrations [647].

However, surface modification of IONPs can be an advantageous approach to reducing their toxicity effectively. For instance, a study investigated the impact of different sizes and coatings on IONPs. Researchers synthesized bovine serum albumin (BSA)-coated IONPs with their PEG derivatives and examined their stability and biocompatibility compared to their counterparts. It was found that BSA-coated IONPs exhibited improved stability and biocompatibility compared to uncoated IONPs, which triggered oxidative stress and caused DNA damage. Moreover, the PEG derivatives of BSA-coated IONPs demonstrated even greater stability and reduced cytotoxicity, highlighting the potential for PEGylation to enhance the protective effects of BSA coatings. In the end, coating applications, specifically with BSA and PEG, can be considered crucial for optimizing the properties of IONPs to reduce toxicity in further studies [648]. 

Additionally, reducing toxicity with pegylation is a commonly employed technique for both inorganic NPs and carbon-based NPs. Similar to inorganic NPs, carbon-based nanomaterials possess potential toxicity that needs to be considered. Like the general factors that influence NP toxicity, type, shape, size, and the surface of the carbon material determine the level of toxicity [649]. Similar to iNP toxicity mechanisms, carbon-based nanomaterials can induce ROS synthesis, DNA and mitochondrial damage, and inflammation.

Even though GO possesses a wide range of applications as a reliable nanomaterial, there are certain long-term drawbacks. One of the main drawbacks of the application of GO is the risk of toxicity. Graphene and graphene-based materials, including graphene NPs, can potentially possess significant toxicity based on the type, synthesis methods, layers, size, and the carbon ratio of the graphene [650]. Depending on the size (higher toxicity in smaller sizes), both graphene and GO can exhibit toxicity through different mechanisms. To make it clear, it was emphasized that graphene can induce acute toxicity and decrease cellular survivability rates; meanwhile, GO can generate ROS and cause DNA damage [651].

Since the surface of graphene and graphene-based NPs is open to modification, many functionalization approaches have been studied to increase biocompatibility and decrease toxicity. For instance, enhancing the capability of GO with polyethylene glycol (PEG) is widely discussed in the current literature, similar to iNP toxicity research. It has been mentioned that the functionalization of GO with PEG strengthens the aqueous stability of the molecule and enhances the biocompatibility and drug delivery activity [652]. A study demonstrated pH-sensitive drug delivery by PEG-functionalized GO, successfully showing enhanced drug delivery with increased compatibility and positive pH-dependent drug release [653]. The toxicity factor was also considered and compared with unfunctionalized GO. Although both types of GO showed dose-dependent toxicity, PEG-modified GO displayed significantly less toxicity. The researchers indicated that the covered surface of GO by PEG could be the main factor behind the results. 

Despite the wide-ranging applications of fullerenes, certain studies indicate their potential toxicity. It has been demonstrated that C_60_ can generate ROS, which are involved in lipid peroxidation and lead to necrotic cell death [654]. Additionally, certain models indicate different mechanisms behind the potential toxicity of C_60_ at increased concentrations. For instance, the toxicity of C_60_ has been investigated in a common model organism, Daphnia magna, due to its significant sensitivity to toxicity, which shows observable changes [655]. The toxicity of C_60_ NPs was tested by administering them to D. magna at different concentrations and compared with titanium dioxide NPs [656]. It was demonstrated that filtered C_60_ exhibited a concentration-dependent mortality ratio in D. magna. Several ppb concentrations were tested during the experiment, ranging from 40 to 880. With a few exceptions, the mortality ratio increased with the ppb concentrations. The lowest mortality ratio was observed at 40 ppb, which was 12%, while the mortality rate reached 100% at 880 ppb. Compared to C_60_, titanium dioxide required higher concentrations to reach C_60_’s mortality levels. A more recent study demonstrated the molecular mechanism of C_60_’s toxicity in D. magna [657]. Certain parameters of D. magna were observed under C_60_ NP treatment at various concentrations. The frequencies of hopping and heart beating were greatly affected. Both frequencies slightly increased at all concentrations, potentially an unintentional response to the initial C_60_ treatment. Over time, both frequencies dramatically decreased based on the concentration. At higher concentrations, C_60_ also negatively affected reproductivity and total population, indicating potential reproduction toxicity. Later, transcriptomic analyses revealed the mechanisms behind the toxicity by showing suppression of the genes responsible for protein synthesis, cell cycle, and energy metabolism. Some researchers also demonstrated that C_60_s could be rapidly ingested during treatment, causing oxidative stress in the same model, thus leading to gut impairments and inhibition of digestive enzymes [658].

In terms of C_60_’s health risk to humans, its investigation is less common compared to different models used for toxicity studies. Therefore, many reviews underline that a considerable conclusion on C_60_ toxicity cannot yet be made [659,660]. This is not unusual considering the applications of fullerene NPs. Additionally, C_60_ might be one of the carbon-based nanomaterials that possess the least toxicity potential. This was highlighted in a comparative study between C_60_, graphene, GO, and carbon nanotubes [661]. Among these nanomaterials, C_60_ showed the least toxic effects on tested microalgae. In addition, C_60_ was the only nanomaterial that did not agglomerate with microalgae cells in seawater. The same research tested these materials on different types of microalgae [662]. While C_60_ showed the lowest toxic levels compared to other materials, its toxic levels also decreased over time, along with graphene. Moreover, there was no trace of a negative effect on microalgae treated with graphene and C_60_ after one week.

It has been known that among carbon-based nanomaterials, carbon black is considered one of the most toxic. This toxicity arises from its potential to cause respiratory issues when inhaled, particularly due to its small particle size, which allows for it to penetrate deeply into the respiratory tract. Furthermore, there is also growing evidence suggesting CBNPs may adversely affect cardiovascular health [663].

Regarding these, researchers conducted a detailed examination of CBNPs, along with TiO_2_ NPs, to investigate their similarities and differences in terms of potential toxicity mechanisms. The study focused on how these NPs interact with proteins and form a complex called a protein corona, which influences their aggregation, cellular uptake, and biological reactivity in physiological environments. They identified several pathways through which CBNPs and TiO_2_ NPs induce toxicity, including enzyme inhibition, disruption of the cytoskeleton, modulation of immune responses, and activation of cellular signaling pathways. Accordingly, it was emphasized that these interactions can lead to substantial cellular changes, impacting various physiological processes and potentially contributing to adverse health effects associated with NP exposure [664].

In another study, the effects of CBNPs exposure on HUVECs and vascular function were investigated. Findings revealed that CBNP exposure induced dose-dependent ROS production in HUVECs, accompanied by increased expression of endothelial adhesion molecules VCAM-1 and ICAM-1. Furthermore, CBNPs altered vascular responses in aortic and mesenteric artery segments, showing complex effects on endothelium-dependent and independent vasorelaxation. These findings collectively underscore CBNPs’ capacity to impair vascular function and endothelial cell integrity, with implications for cardiovascular health in environmental and occupational settings [665].

Additionally, four commercial CBNPs were evaluated for their toxicological impact on both lung and knee joint health. Utilizing a combination of *in vivo* mouse models and *in vitro* cell culture assays, they identified significant differences in toxicity profiles among the samples. Inhalation exposure to CBNPs induced lung injuries marked by alveolar collapse, hyperemia, and inflammatory responses, varying in severity depending on the NP type. Moreover, CBNPs triggered pro-inflammatory responses in macrophages and activated chondrocytes in knee joints, potentially exacerbating joint disorders like osteoarthritis and rheumatoid arthritis [666].

Similar to most of the iNPs and CBNs, CQDs also exhibit toxicity potential at high concentrations both *in vivo* and *in vitro* [667]. In most of these models, increased levels of oxidative stress caused by CQDs are mentioned. A study investigated the toxic effects of CQD on the gut–liver axis and gut microbiome in Cyprinus carpio [668]. CQD exposure after 5 weeks induced oxidative stress, leading to an inflammatory response and damage to the intestines and liver. Similar induction of oxidative stress by CQD was demonstrated in algae [669]. A different mechanism of cellular toxicity by CQDs was observed in *E. coli* [670]. The accumulated CQDs significantly altered osmotic pressure and surface charges, and induced lipid peroxidation, but only at high concentration and exposure times. 

However, the toxicity of CQDs can be controlled by modifying the material. One strategy to alter the toxicity is doping CQDs with certain elements. The structural change from doping can reduce toxicity to extremely low levels. For instance, the non-toxicity of nitrogen-doped CQDs was demonstrated in both *in vitro* and *in vivo* models [671]. HeLa cells were treated with nitrogen-doped CQDs at certain concentrations, between 6.5 and 400 μg/mL. Cell viability tests revealed no trace of any toxicity after 120 h, even at the highest concentrations. Moreover, cell apoptosis analysis showed that nitrogen-doped CQDs did not induce apoptosis or interfere with any of the cell cycles in HeLa cells. Subsequently, the CQDs were tested on Swiss albino rats. Various parameters of the rats were analyzed after CQD treatment. It was demonstrated that CQDs did not alter the weight of the liver and kidneys, protein content, or oxidative stress enzymes compared to the control group. Hematological analysis supported the non-toxic effects of the CQDs. Additionally, a comparison study between undoped CQDs and two types of doped CQDs, N and folic acid, was conducted to compare their toxicity [672]. *In vitro* cytotoxicity assays (L929, C6, and normal cell MDCK) revealed that none of the CQDs induce any toxicity or changes even at high concentrations (1 mg/mL). An *in vivo* study on mice was also conducted. Histological analysis showed that all CQDs did not induce any significant toxicity in several organs.

There is increased research on modifying materials that pose toxicity risks, especially NPs with wide-ranging applications. Fortunately, most of these modifications can reduce the toxicity to desired levels. Thus, toxicity studies on modified NPs, especially comparison studies, carry significant importance. Obtaining a less toxic version of an NP is not sufficient. Further studies need to be conducted to confirm the activity of the NP, ensuring it can still be applied in the targeted area.

Organic NPs represent the safest type of NPs in applications due to their naturally occurring substantial biodegradability. However, some issues related to the potential toxicity of organic NPs have been addressed. For instance, it was stated that when the organic NPs are predominantly utilized in food studies are considered, there are certain proposals that the role of these NPs in the gastrointestinal regions should be investigated [673]. Discussions exist on the potential toxicity of certain subtypes of organic NPs and factors that require consideration. The potential toxicity of chitosan NPs has been discussed, using the zebrafish model in extreme doses, with contradictory results [674]. Additionally, based on the application of chitosan NPs in cancer research, it has been shown that the toxicity levels are either very low or non-existent in normal cells and zebrafish embryos [675]. This comprehensive review also points out the necessity of further studies to carry out the application of chitosan NPs in human studies.

Organic NPs, particularly those consisting of fats, have gained significant attention due to their extensive use in drug delivery systems. These NPs, as mentioned earlier, are designed to encapsulate and transport therapeutic agents effectively, thereby improving bioavailability and enabling targeted delivery to specific tissues or cells. However, some aspects require taking into account.

Although LBNPs are considered non-toxic, in some circumstances, exposure to higher doses can lead to adverse outcomes. For instance, studies have shown that prolonged exposure of LBNPs may induce toxic effects. In line with this, researchers investigated sub-acute toxicity of SLNs in mice models over a 10-day period. They assessed the biocompatibility of two formulations, natural wax- and tristearin-based, through a combination of *in vitro* hemolysis tests and *in vivo* analyses. The results indicated that while SLNs induced significant inflammation in adipose tissue and fat deposition, there were negligible signs of toxicity [676].

Organic NPs demonstrate non-toxic characteristics thanks to their extreme biodegradability and high bioavailability. They are considered safe for many applications. However, their NP nature should be considered. Many properties of NPs, especially physicochemical attributes, shape the toxicity potential of these particles [677]. Today, it cannot be completely asserted that organic NPs are exempt from this issue. Still, based on the majority of the literature, it is clear that organic NPs do not possess significant toxicity, especially compared to other types of NP. Investigation of these physicochemical attributes can hasten the development of non-toxic organic NPs. These particles exhibit distributed sizes, and some preparation methods can even create larger particles, which goes outside the general definition of an NP [678]. Hence, detailed consideration of the size of the NPs, especially for organic NPs, can ease the path to the enhancement in applications and commercialization of NPs without the risk of toxicity. 

The size of NPs is important in their manufacture since it influences many properties and uses. The substantial surface area per volume of NPs, along with size, affects several size-dependent phenomena such as chemical, electrical, magnetic, and mechanical properties. For example, smaller NPs have a larger surface-area-to-volume ratio, which improves reactivity and interactions with other materials. This is important in catalysis, drug delivery, and monitoring. Size influences optical qualities, including color and fluorescence, which are useful in biological imaging and diagnostics. Magnetic features, such as superparamagnetism exhibited in certain size ranges, are significant for MRI and data storage. Nanoscale quantum effects have advantages in electronics, photonics, and quantum computers [679].

Mechanical qualities, including strength and elasticity, are considerably altered, facilitating the development of complicated substances such as nanocomposites. Size determines cellular absorption, biodistribution, and toxicity in biological contexts; hence, smaller nanoparticles are suitable for targeted medication delivery and medical imaging. For functioning to continue, size-dependent stability and aggregation are essential. By regulating the size of NPs, catalytic activity is increased and more active sites for reactions are available. Furthermore, smaller NPs diffuse and move more effectively, which is crucial for medicine delivery and environmental cleanup applications. As a result, the size of NPs has a fundamental impact on their chemical, biological, and physical characteristics, which influences their applicability and performance in a variety of domains [680].

Navigating numerous regulatory concerns is necessary for the commercialization of NPs, and these create substantial obstacles. Complex and regionally specific regulatory regimes for NPs necessitate comprehensive safety and efficacy testing to meet strict standards. The environment and public health are protected from NPs by these rules. Nonetheless, the absence of defined procedures and the dynamic character of nanotechnology can cause ambiguities and hold up approval procedures. Due to the importance of trade in nanoparticles on a worldwide scale, new international organizations have been founded to share responsibilities in this field, such as the International Council on Nanotechnology (ICON) and the International Organization for Standardization (Geneva, Switzerland). The National Nanotechnology Initiative (NNI) was founded in the United States of America in 1996 to coordinate the development of nanoscience and technology across multiple government agencies, including the Environmental Protection Agency (EPA), the Food and Drug Administration (FDA), the Department of Labor through the Occupational Safety and Health Administration (OSHA), and the National Institute for Occupational Safety and Health (NIOSH) [681].

## 5. Conclusions 

NPs have a diverse range of applications with significant and promising results. According to the classification of these particles, there are similar areas of applications with shared goals, and also specific applications with utilization of unique properties. The base material certainly shapes the application and activity efficiency of the NPs, and these distributed applications are the primary examples of this. Even though there is much research in NP application, it is clear that some of these areas are predominantly occupied by certain types. For instance, organic NPs are mainly involved in food packaging as non-toxic and biodegradable materials, and in preservation studies thanks to their favorable structure for encapsulation. On the other hand, carbon-based NPs are widely used in industrial applications due to their unique physical properties, rather than in biological applications, as some materials possess great toxicity potential. Meanwhile, inorganic NPs are seen in antibacterial and bioimaging studies. Concurrently, some of these areas are common among all three types of NPs due to the shared characteristics of the NP structure and the hybridization of these materials. Yet, the number of studies conducted in these shared areas needs to be considered as well. For instance, even though all three classes have been used in drug delivery systems, it is evident that inorganic and organic materials are predominantly applied compared to carbon-based NPs. Nanotechnology expresses accelerated development and disruption in many areas. In particular, there is a great deal of promise for using nanoparticles as medication delivery methods in the future. With the help of NPs, medications can be precisely delivered to specific tissues or cells, minimizing adverse effects and improving the effectiveness of treatment. These developments could revolutionize healthcare and greatly enhance patient outcomes. Thus, comprehending the effect of these materials on NP activity is crucial, and will be informative for future developments in NP application.

## Figures and Tables

**Table 1:** NPs according to their highlighted properties and application areas.

Type of the NP	Effective Properties	Area of Application	References
Magnetic NP	- Superparamagnetic - Heating efficiency - Magnetic susceptibility	- Magnetic resonance imaging (MRI) - Hyperthermia therapy - Drug delivery	[24,97,98,99]
Ceramic NP	- High mechanical strength - High stability - Good body response - Resistant to temperature and pH - Flexibility in terms of administration methods (oral, inhalation, etc.) and ease of integration into hydrophobic and hydrophilic systems	- Drug delivery - Tissue engineering - Biosensors and diagnostic - Drug carrier	[32,34,35]
Semiconductor NP	- Antimicrobial activity - Luminescence	- Dental implants - Rubber industry - Food packaging - Cancer	[36,38,100,101,102]
Graphene NP	- Thermal conductivity - Preferred mechanical and optical properties (strong supportive material) - Hybridization potential with metal ion NPs	- Drug delivery - Antibacterial - Anticancer - Industrial	[56,103,104,105]
Fullerene NP	- Highly antioxidant - UV-induced damage protection	- Dermatology - Drug delivery - Diabetes - Antioxidant - Antitumor	[70,106,107,108]
Carbon black NP	- Electrical conductivity - High reinforcement capability - Pigmentation - High surface area	- Reinforcing agents in the rubber industry - Conductive additives in electronics - Manufacture of cement-derived composites	[75,109,110]
Carbon quantum dots	- Photoluminescence - Chemical stability - Versatile surface functionalization - Antibacterial activity	- Bioimaging - Drug delivery - Fabrication of sensors - Antibacterial research	[85,94,111]
Chitosan NPs	- Mucoadhesive - Antimicrobial - Open for modification (including metal ions)	- Drug delivery - Food packaging and preservation - Wound healing - Diabetes	[112,113,114,115,116]
Alginate NPs	- pH-sensitive - High chemical modification choices - Compatible with other polymers - Utilized for nanogel fabrication	- Drug delivery - Food preservation - Diabetes	[117,118,119]
PLA/PLGA NPs	- Compatible with other polymers - Significant biodegradability (CO_2_ as end product) - Sustained drug release	- Drug delivery systems - Imaging - Diabetes - Tissue engineering - Wound healing	[120,121,122,123,124]
Liposome NPs	- Encapsulation of both hydrophilic and hydrophobic molecules - Controlled and targeted delivery of drugs - Ease of functionalization	- Drug delivery - Cosmetics - Food preservation - Wound healing - Gene delivery	[92,125,126,127]
Solid-lipid NPs	- Encapsulation of both hydrophilic and hydrophobic molecules - Protection of labile drugs - Enhanced physical stability	- Drug delivery - Cosmetics - Food preservation - Cancer	[128,129,130,131]
Nanostructured lipid carriers	- High drug loading capacity - Enhanced drug stability - Controlled drug release - Preventing drug expulsion	- Drug delivery - Cosmetics - Food preservation - Cancer	[132,133,134,135]
Carbohydrate-based NP	- Biocompatible - Biodegradable - Functional flexibility	- Drug delivery - Cancer - Food preservation	[117,136,137,138]
Protein-based NP	- Biodegradable - High yield and entrapment efficiency - Suitable for the design of oral and topical drug carriers - Self-assembling features	- Drug delivery - Vaccine carriers - Bioimaging - Therapeutic protein delivery - Nucleic acid (DNA, RNA, and miRNA) delivery	[139,140,141]

## Data Availability

Not applicable.
